# Dysbiosis and Variation in Predicted Functions of the Granulation Tissue Microbiome in HPV Positive and Negative Severe Chronic Periodontitis

**DOI:** 10.1155/2019/8163591

**Published:** 2019-04-14

**Authors:** Rebecca Chowdhry, Neetu Singh, Dinesh Kumar Sahu, Ratnesh Kumar Tripathi, Archana Mishra, Anjana Singh, Indrashis Mukerjee, Nand Lal, Madan Lal Brahma Bhatt, Ravi Kant

**Affiliations:** ^1^Department of Periodontology, King George's Medical University, Lucknow 226 003, India; ^2^Department of Molecular Biology, Center for Advance Research, King George's Medical University, Lucknow 226 003, India; ^3^Imperial Life Sciences, 463 Phase City 2, Sector 37, Gurgaon, Haryana 122001, India; ^4^Department of Thoracic Surgery, King George's Medical University, Lucknow 226 003, India; ^5^Department of Pulmonary and Critical Care Medicine, King George's Medical University, Lucknow 226 003, India; ^6^Department of Pharmacology, King George's Medical University, Lucknow 226 003, India; ^7^Vice Chancellor, King George's Medical University, Lucknow 226 003, India; ^8^Director, All India Institute of Medical Sciences, Rishikesh 249 203, India

## Abstract

Retrospective analysis has already shown correlation between severe Chronic Periodontitis (CP) cases with human papiloma virus (HPV). Hence, we aimed to explore deep-seated infected granulation tissue removed during periodontal flap surgery procedures for residential bacterial species between HPV+ and HVP- CP cases, which may serve as good predisposition marker for oral cancer. All CP-granulation samples showed the prominence of* Firmicutes, Proteobacteria*, and* Bacteroidetes* phyla with an abundance of gram negative anaerobes, except* Streptococcus*. In Beta diversity nonmetric multidimensional scaling plot, the random distribution of species was observed between HPV+ and HPV- CP granulation-samples. However, an abundance of* Capnocytophaga ochracea* was observed in HPV+ CP samples (p<0.05), while* Porphyromonas endodontalis*,* Macellibacteroides fermentas*,* Treponema phagedenis*, and* Campylobacter rectus* species were highly abundant in HPV- CP samples (p<0.05). The differential species richness leads altered functions related to mismatch-repair and nucleotide excision-repair and cytoskeleton-proteins. Hence, differential abundance of gram negative bacterial species between HPV+ and HPV- granulation-samples under anaerobic conditions may release virulence factors which may alter pathways favouring carcinogenesis. Hence, these species may serve as good predisposition marker for oral-cancer.

## 1. Introduction 

Chronic periodontitis (CP), bacterially induced chronic inflammatory disease of the periodontium, is one of the 50 most prevalent disabling health conditions affecting up to 80-90% of the population worldwide and in India [1, 2]. It is the main cause of tooth loss in adults and directly linked to systemic chronic diseases, including cardiovascular disease, diabetes, stroke, and preterm birth pneumonia and cancer [3, 4]. Risk-factors like human-papilloma-virus (HPV), smoking, and tobacco further complicate the problem. The primary etiology of periodontitis disease is plaque biofilm affecting the periodontal tissues. Pocket formation, loss of periodontal attachment, loss of alveolar bone, root furcation exposure, tooth mobility in advanced cases of bone destruction, and infected granulation tissue formation are the main features of CP. During the periodontal flap surgery, the removing of deep seated pocket granulation tissue is the main process of treatment for filling wounds and improving the condition of new attachment.

Currently a total of 600 bacterial species are represented as periodontal pathogens that can colonize dental surfaces over (supra) and below (sub) the gingival margin and oral mucous membranes [3, 5]. Most evaluated, subgingival plaque samples from CP patients possess both periodontal pathogens (including anaerobes and facultative aerobes) [3] and commensal flora (*actinomyces*,* streptococcus*, and* staphylococcus* sp.). Dominance of periodontal pathogens mediated chronic inflammation may cause deregulation of local oral immune response. This may provide advantage to opportunistic infections which may predispose to carcinogenesis. Other sources like saliva and pooled and site-specific subgingiva have also been profiled for microbial flora. Presence of periodontal pathogens was observed in stimulated saliva and pooled subgingival plaque samples [6]. Based on above studies, we comprehend that most of the sites affected by CP have been profiled although deep-seated infected granulation tissue removed during periodontal flap surgery procedure has not been profiled for its microbiota. Importantly, granulation tissue has been suggested as a source of stem cells-involved in filling wounds and bone regeneration [7].

Additionally, deeper-pockets and higher mean alveolar bone loss mediated-CP have been shown to be associated with HPV-16 positive compared to negative base-of-tongue-SCC samples (3.90 mm vs. 2.85 mm, P = .01). Recent reports have also compared the abundance of microbial species between normal and oral cancer samples in significant loss of bacteria and have been observed in head and neck squamous cell carcinoma compared to control in saliva samples. They also evaluated HPV status which showed slight discrimination through beta dispersion with significant higher variants in OPSCC HPV+ samples compared to control and OSCCC HPV- samples (p<0.02) [8].

Different sequencing based methods have been used to explore the microbial complexity of CP samples. However, most recent publications suggest long read sequencing technology (V1-V9 full length variable region of 16s ribosomal gene) can provide finer phylogenetic profiling [9].

Hence, we hypothesized assessing association of microbiological profile with HPV+ and HPV- CP cases (Clinical Attachment Loss = or > 5mm) through long read sequencing technology [9]. We identified differences between HPV+ and HPV- CP bacterial communities' at all taxonomic levels. Additionally, we were able to identify least expressed uncultured bacterial species at genera and species level. Major shifts at the level of genera in HPV+ versus HPV- CP samples may provide a basis for further understanding HPV microbe interactions and creation of an inflammatory niche in CP which may lead to the process of carcinogenesis.

## 2. Materials and Methods

### 2.1. Screening of Patients, Sample Collection and DNA Extraction

A total of 125 CP patients were screened and 40 patients between age of 18 and 66 years (average 37±11 years) with the history of no smoking and tobacco chewing, bleeding on probing, Periodontal pockets = or > 5mm, and having Clinical attachment loss (CAL) were included in the study (Supplementary Table 1). Patients with the history of smoking, tobacco chewing, systemic disorders (diabetes, hypertension etc.), blood dyscrasias, compromised immune system, consumption of antibiotics in the past three to six months, and surgical procedures in the past three to six months were excluded from the study. The study protocol was approved by the Institutional Review Board of the King George's Medical University. Written informed consent was obtained from all patients.

After clinical examination following diagnostic parameters as reported in [10] were subjected to routine scaling and root planing [11]. After strict oral hygiene for one week, i.e., cessation of any deleterious habit, completely periodontal flap surgery was conducted in 40-CP cases. Granulation tissues were collected during flap surgery and processed for DNA extraction using Qiagen mini DNA isolation Kit. DNA recovered from the extraction process was quantified using Q5000 UV-Vis Spectrophotometer (Quawell San Jose, CA) and Qbit Fluorimeter and stored at -20°C for further analysis.

### 2.2. Detection of HPV-16 E6

PCR was performed for detection of HPV in all 40 CP samples using HPV 16* E6 *and* B*-actin specific primers; HPV-16* E6 *forward primer, 5'- TCAGGACCCACAGGAGCG-3'; HPV-16* E6 *reverse primer, 5'-CCTCACGTCGCAGTAACTGTTG-3' and* B*-actin forward primer, 5'-TCACCCACACTGTGCCCATCTACGA-3',* B*-actin reverse primer, 5'- CAGCGGAACCGCTCATTGCCAATGG-3' in triplicate [12]. Additionally, we pooled six HPV+ oral cancers and treated them as a positive control for detection of HPV.

### 2.3. 16S rRNA Gene Amplification, Multiplexing, and PacBio Sequencing

As per [10], (a) amplification of full length 16S ribosomal gene (1464 bp) from 50 ng/*μ*l DNA from each CP sample using V1-V9 variable region primers; (b) barcoding of each amplified amplicon using paired 16-nt symmetric barcodes (https://github.com/PacificBiosciences/Bioinformatics-Training/wiki/Barcoding-with-SMRT Analysis-2.3) (Table 1); (c) multiplexing of barcoded-purified amplicons in a group of 5 libraries (Supplementary Table 2); and (d) Sequencing using DNA Sequencing Reagent Kit 4.0 v2 (Pacific Biosciences, USA) in a total of 24 SMRT cells (3 cells/library) using 6-hour movies data collection protocol were performed.

### 2.4. Raw Sequence Analysis and Metagenome Functional Predictions

The PacBio generated raw sequences were processed for RS_Read of Insert (ROI) algorithm, demultiplexed, processed, aligned, and classified at species level and generated alpha (Ace, chao, shannon index, invsimpson and sobs) and beta diversity (jclass, thetayc, nmds) indexes using Mothur (version 1.34.4) [13] against “Greengenes reference database” gg_13_8_99 as per [10]. Rarefaction curves, principal coordinates (PCoA), and nonmetric multidimensional scaling (NMDS) were also generated using PASTv3.11 [10, 14].

Raw data analysis and metagenome functional predictions were performed as per [10]. Briefly, metagenomics functional inferences were processed through PICRUSt tool [15] using marker gene data and database of reference genomes from the 16S rRNA data and KEGG (Kyoto Encyclopedia of Genes and Genomes) database [16]; grouping of closed-reference OTUs (97% sequence identity) was performed using uclust and the greengenes reference database; OTUs abundance was normalized automatically using 16S rRNA gene copy numbers from known bacterial genomes in Integrated Microbial Genomes (IMG) [17]; predicted genes and their function were aligned to KEGG database; differences between the abundance of functional pathways and the species abundance among groups (HPV+/HPV-) were compared through software STAMP [5]; statistics and visualization of functional data like heat maps, square and PCA plots were generated displaying differences between the groups using STAMP; two-side Welch's t-test and Benjamini-Hochberg FDR correction were used in two-group analysis.

## 3. Results

### 3.1. Multiplexed Amplicon Sequencing of Full-Length 16S rRNA Gene

A total of 8 pooled and barcoded 16S rRNA amplicon libraries were prepared and sequenced on PacBio RSII system. Following sequencing and demultiplexing using Read of Insert protocol (RS_Read of Insert), 3 samples (CP25, CP28, and CP 29) were excluded from further analysis due to less number of reads. Following removal of 3 samples, a total of 3,59,296 raw CCS reads were generated from 37 CP samples with an average accuracy of 99.36%, 12 numbers of mean passes, and a mean length of 1,402 nt. Further, removal of chimeras and CCS reads shorter than 1400 nt and longer than 1600 nt and 2,72,221 reads were obtained from 37 CP samples (Supplementary Table 3). The number of processed full-length 16S rRNA sequences per CP sample ranged from 672 to 28,780 with an average of 7,357 reads/sample (±SD 5117 reads) (Supplementary Table 3).

### 3.2. Assessment of Severe CP Associated Bacterial Diversity in Granulation Tissue Isolated during Periodontal Flap Surgery Using High-Coverage PacBio 16S rRNA Gene

#### 3.2.1. Study Group Characteristics

In the present study, a total of 37 CP samples were collectively analyzed to profile the bacterial communities in unexplored granulation tissue of severe CP cases. The numbers of operational taxonomic units (OTUs) at a 3% dissimilarity level and the diversity estimates are listed in Supplementary Table 4. To assess the diversity of bacterial community present within each sample, a series of alpha diversity indices were calculated. Alpha-diversity measures the biological diversity of a community, taking both species richness and variance in species proportion into consideration. Using Shannon index as the metric for alpha-diversity, we found the average to be 6.84 (±SD 0.60) with individual samples diversity index ranging from 2.19 to 8.05 (Supplementary Table 5a). Alpha-Diversity Analysis tests ANNOVA (Repeated Measures ANNOVA) showed no significant differences between samples equal means (Supplementary Table 5b). Tukey's pairwise test also showed no significant difference between sampled species in each sample (Supplementary Table 5c). Alpha-Diversity Analysis using MOTHUR generated rarefaction curves which nearly plateau off for the majority of the samples (Supplementary Figures 1a and 1b), indicating that sufficient sampling has been performed to capture the total diversity of the communities.

### 3.3. Determination of Phyla in CP Samples Identified in Tissue Samples

We found a total of 13 assigned phyla present with 3 of these dominating across all of the samples:* Proteobacteria* (31.43%),* Firmicutes* (31.32%), and* Bacteroidetes* (22.08%). Next* Spirochaetes, Actinobacteria*, and* Fusobacteria* showed 2.59, 3.28 and 8.53%, respectively, and other six phyla had a relative abundance lower than 1% (Supplementary Figure 2a).

### 3.4. Determination of Species/Phylotypes/Genus Level in CP Granulation Tissue Samples

The observed number of OTUs based on high-quality sequences processed through Mothur package against green gene_13_8_99.gg.tax database for species richness in 37 CP samples. Among these a total of 112 observational IDs were unclassified and 125 were classified at the species level (Supplementary Figure 2b).

In* Firmicutes*,* Streptococcus* showed dominance (42.5%) with unclassified* Streptococcus* ID1000186 sp. (86.9%) and* Streptococcus agalactiae* (12.0%) rest were less than 1%. Both* Veillonella* and* Selenomonas* showed 19.2% and 18.9% OTUs. Two species* parvula* and ID1004529 of* Veillonella* showed a predominance of 45.2 and 54.67 OTUs, while* Selenomonas bovis*,* Selenomonas* ID1030589, and* Selenomonas noxia* showed OTUs ranging from 21.39 to 40.62 (Supplementary Figure 2b).

In* Proteobacteria*,* Neisseria* showed dominance with unclassified* Neisseria* ID1007399 sp. 35.2% and* Neisseria oralis* 62.61%, and the rest were less than 1%. Both* Campylobacter* and* Sphingomonas* had 8% OTUs individually (Supplementary Figure 2b).

In* Bacteroidetes*,* Prevotella* showed dominance (43.7%) with unclassified Prevotella ID1000563 sp. (51.2%),* Prevotella intermedia* (20%), and* Prevotella nigrescens* (12.59%). Next dominance was of* Capnocytophaga* (27.9%) with unclassified* Capnocytophaga ochracea* sp. (67.8%) and* Capnocytophaga* ID1001498 (31.9%). 14.41 % was the occurrence of* Macellibacteroides fermentans* while* Porphyromonas* with two species* endodontalis* and ID1001120 showed 4.0% richness and* Tannerella*- ID101201 5.6% (Supplementary Figure 2b).

### 3.5. Comparisons of Microbial Community Profiles CP Granulation Tissue Samples between HPV+ and HPV- Samples and Associated Functions

Generated OTUs through Mothur were further analysed between HPV+ and HPV- CP samples. OTU based correlation coefficient analysis showed R^2^ =0.82 between HPV+ and HPV- samples depicting a poor relationship between microbial abundance between the two groups (Supplementary Figure 3a). Further, nonmetric multidimensional scaling and principal component analysis of samples represented OTUs between HPV+ and HPV- samples which were similarly based on distances and showed random distribution (Supplementary Figures 3b and 3c). Different beta-diversity analysis indexes reporting beta diversity between HPV+ and HPV- CP dataset are presented in Supplementary Table 6.

**Figure 1:**
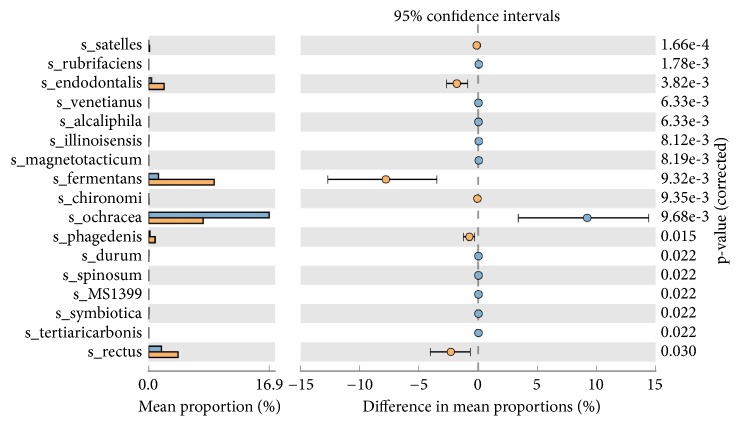
Mean proportion of bacterial species abundance between HPV+ (blue bar) and HPV– (yellow bar) CP samples. The significant differences observed between the two groups at 95% confidence level and p<0.05 are reported.

STAMP based generation of heat-map of OTU's showed, among the HPV+ samples, CP-15 (55 yrs-F), CP-19 (32 yrs-F), CP-1 (26 yrs-F), and CP-24 (39 yrs-F) were in one cluster while CP-5 (26 yrs-F), CP21(33 yrs-F), and CP-26 (48 yrs-M) showed individual existence (Supplementary Figure 4). PICRUST analysis of normalized OTUs showed the formation of three distinct clusters among the HPV+ samples. Among the HPV+ samples, high and significant (p<0.05) abundance of* Bacteroidetes* phyla-*Capnocytophaga ochracea* was observed, while* Bacteroidetes* phyla-*Porphyromonas endodontalis*,* Macellibacteroides fermentas*, and* Spirochaetes* phyla-*Treponema phagedenis* and* Protreobacteria* phyla-*Campylobacter* rectus species were highly abundant in HPV- samples (Figure 1).

**Figure 2:**
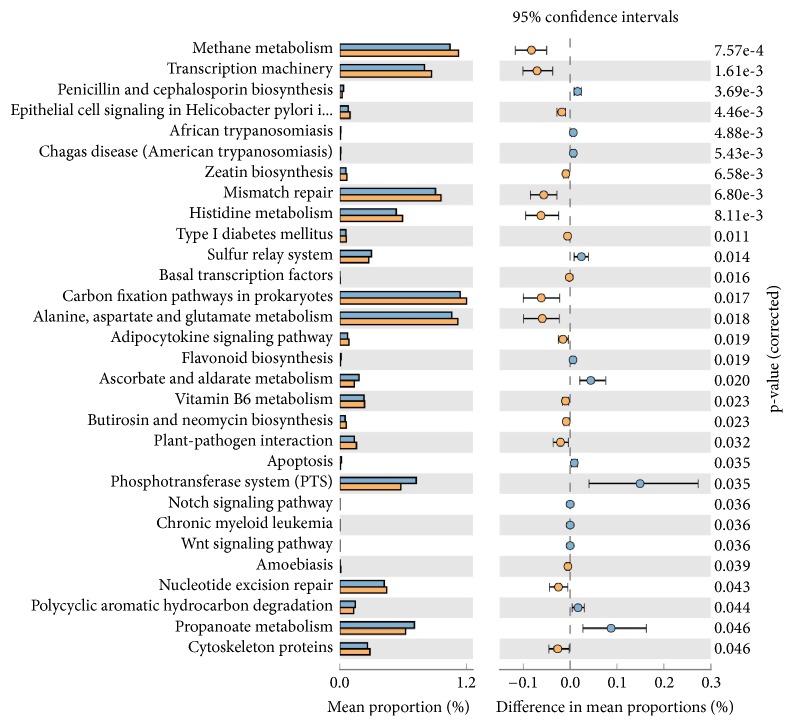
Mean proportion of bacterial species functional predictions between HPV+ HPV+ (blue bar) and HPV– (yellow bar) CP samples. The significant differences observed between the two groups at 95% confidence level and p<0.05 are reported.

Functional predictions of the observed species abundance showed the formation of two distinct clusters HPV+ CP and HPV- CP cases in heat-map profiles (Supplementary Figure 5a), with correlation coefficient analysis R^2^ = 0.99. As per Figure 2, difference in mean proportion ranging 0.0-0.1% included highly significant penicllin and cephalosporin biosynthesis (p=3.69e^−3^) and significant-sulphur relay system (p=0.01), ascorbate and aldarate metabolism (0.02), polyaromatic hydrocarbon degradation (p=0.04), andpropanoate metabolism (p=0.04). Only phosphotransferase system (PTS) showed mean proportion between 0.1 and 0.2% with significance value of p=0.03. Among HPV- sample the difference in mean proportion ranged between 0.0 to 0.1 including highly significant-methane metabolism (p=7.57e^−4^), transcription machinery (p=1.61e ^−3^), epithelial cell signalling in helcobacter pylori (4.66e ^−3^), mismatch repair (p=6.80e ^−3^) histdine metabolism (p=8.11e ^−3^) and significant-carbon fixation pathways in prokaryotes (p=0.017), alanine, aspartate, and glutamate metabolism (p=0.01), plant pathogen interaction (p=0.03), nucleotide excision repair (p=0.04), and cytoskeleton proteins (p=0.04), while propanoate metabolism, sulphur relay system ascorbate, and aldorate metabolism, phosphotarnsferase system (PTS), and polycyclic aromatic hydrocarbon degradation related microbial functions were highly represented in HPV+ samples in comparison to HPV- samples. Among the HPV- samples, a significantly high (p>0.05) prevalence of microbial communities was observed in methane metabolism, transcription machinery, mismatch repair, histidine metabolism, nucleotide excision repair, cytoskeleton proteins, etc. (Figure 2).

### 3.6. Comparisons of Microbial Community Profiles CP Granulation Tissue Samples between Male and Female Samples and Associated Functions

**Figure 3:**
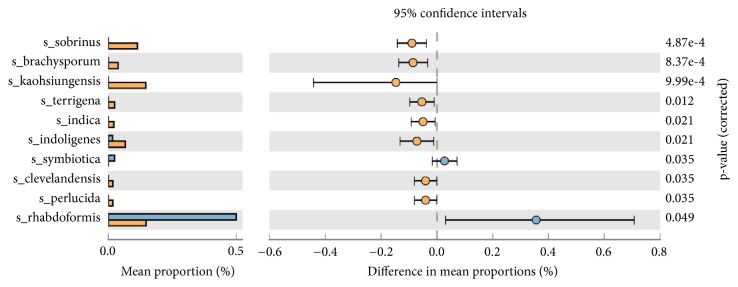
Mean proportion of bacterial species abundance between male (blue bar) and female (yellow bar) samples in CP samples. The significant differences observed between the two groups at 95% confidence level and p<0.05 are reported.

Generated OTUs through Mothur were also analyzed between male and female-CP samples. OTU based correlation coefficient analysis showed R^2^ = 0.96 between male and female-CP samples depicting a poor relationship between microbial abundance between the two groups (Supplementary Figure 6a). STAMP based generation of heat-map of OTUs had, among the male and female-CP, a distributive type (Supplementary Figure 6b). PICRUST analysis of normalized OTUs showed a significant dominance of* Desulfobulbus rhabdoformis* (p=0.05; difference in mean proportion 0.2-0.4%) and serratia symbiotic in males (p=0.04; difference in mean proportion 0.0- (-0.2%) and* Pseudoxanthomonas kaohsiungensis *(p=9.99e ^−4^; difference in mean proportion 0.0-0.2%) in females (Figure 3).

**Figure 4:**
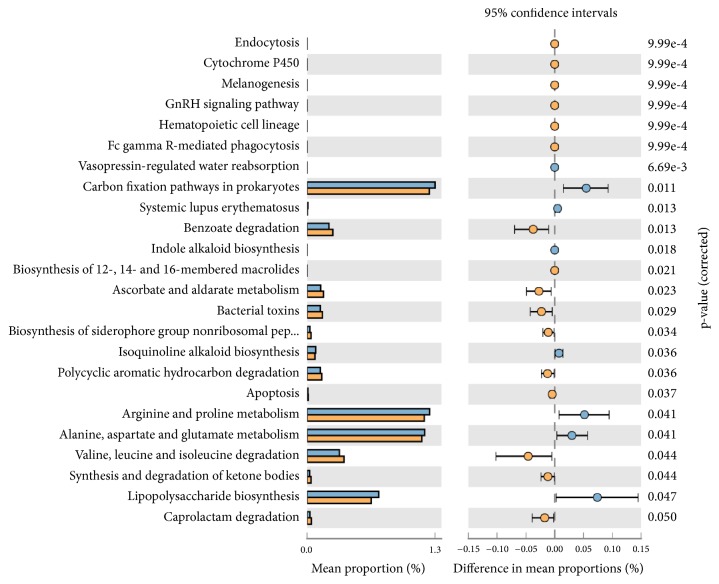
Mean proportion of bacterial species functional predictions between male (blue bar) and female (yellow bar) samples in CP samples. The significant differences observed between the two groups at 95% confidence level and p<0.05 are reported.

Functional predictions of the observed species abundance showed the formation of two distinct clusters (pathway based-y-axis) in heat-map profiles (Supplementary Figure 7a), with correlation coefficient analysis showing R^2^ = 0.998. Difference in mean proportion between male and female (ranged between 0.00 and 0.05) showed high representation of pathways of I cluster-lipopolysaccharide biosynthesis (p=0.05), alanine, aspartate, and glutamate metabolism (p=0.04), arginine and proline metabolism (p=0.04), and carbon fixation pathways in prokaryotes (p=0.11). Among females, a significantly high (p<0.05) prevalence of benzoate degradation (p=0.13), ascorbate and aldorate metabolism (p=0.23), bacterial toxins (p=0.29), valine, leucine, and isoleucine degradation (p=0.44), and synthesis and degradation of ketone bodies pathways (p=0.44) was observed (Figure 4).

### 3.7. Comparisons of Microbial Community Profiles in CP Granulation Tissue Samples between Samples Aged above 40 and below 40 and Associated Functions

Generated OTUs through Mothur were also analyzed between above 40- and below 40-year-old CP patients samples. OTU based correlation coefficient analysis showed R^2^ = 0.96 between above 40- and below 40-year-old CP patients samples depicting a poor relationship between both kinds of microbial abundance between the two groups (Supplementary Figure 8a). Further, beta diversity nonmetric multidimensional scaling plot represented OTUs between above 40- and below 40-year-old CP patient's samples which were similarly based on distances and showed random distribution.

**Figure 5:**
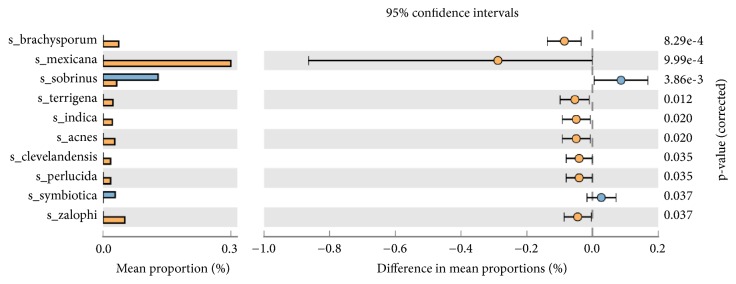
Mean proportion of bacterial species abundance between samples above 40 (blue bar) and below 40 years (yellow bar) age in CP patients. The significant differences observed between the two groups at 95% confidence level and p<0.05 are reported.

STAMP based generation of heat-map of OTUs had, among the above 40 and below 40-year-old CP patient's samples, a distributive type (Supplementary Figure 8b). PICRUSt analysis of normalized OTUs showed a significant dominance of* Streptococcus sorbinus *(p=3.86^e-3^, difference in mean proportion 0.0-0.2),* Serratia symbiotica* (p=0.037, difference in mean proportion 0.0-0.2) in those above 40 years of age, and* Pseudoxanthomonas mexicana* (p=9.99^e-4^, difference in mean proportion 0.0-(-0.4) in those below 40 years of age (Figure 5).

**Figure 6:**
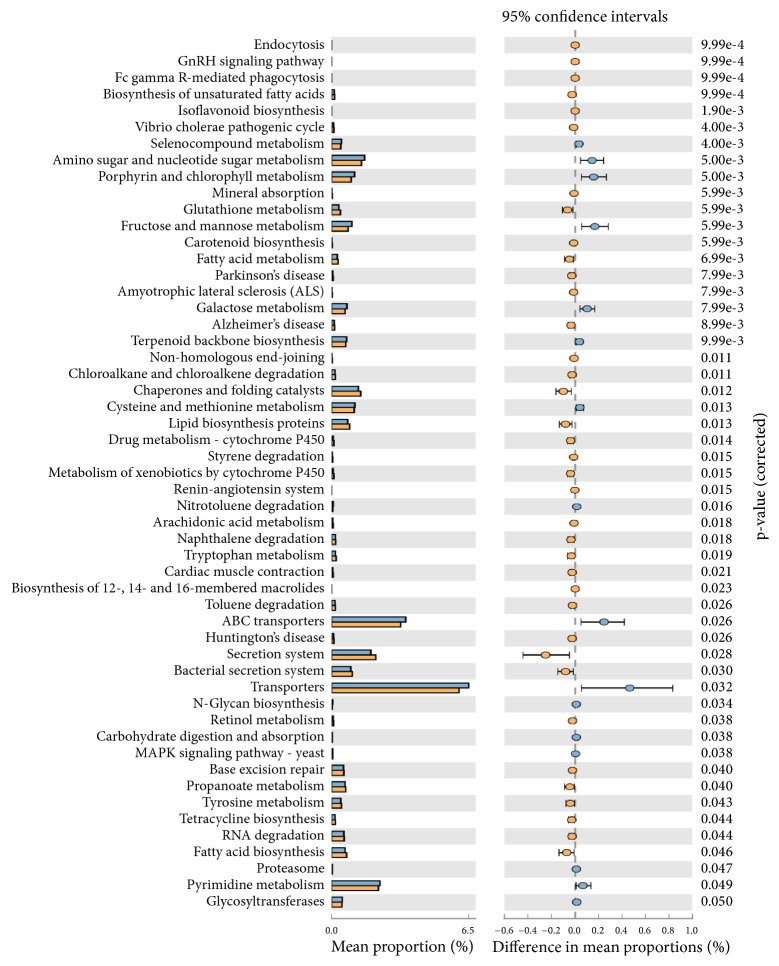
Mean proportion of bacterial species functional predictions between samples above 40 (blue bar) and below 40 years (yellow bar) age in CP samples. The significant differences observed between the two groups at 95% confidence level and p<0.05 are reported.

Functional predictions of the observed species abundance showed the formation of two distinct clusters in heat-map profiles (Supplementary Figure 8c), with correlation coefficient analysis of R^2^ = 0.99. Amino sugar and nucleotide sugar metabolism, porphyrin and chlorophyll metabolism, fructose and mannose metabolism, galactose metabolism, cysteine and methionine, ABC-transporters, and transporters were highly represented in those above 40 compared to those below 40 years of age (p<0.05). Among those below 40 years of age, a significantly high (p>0.05) prevalence of secretion system and bacterial secretion system pathways (p=0.03) was observed (Figure 6).

## 4. Discussion

The recent study on profiling of coral-associated bacterial communities [7] very well demonstrated the application of PacBio sequencing technology for microbial diversity analysis. In the present study, we utilized one-step PCR procedure to amplify 16S gene with bar-coded, HPLC purified primers as it was aimed to amplify only a single target harbouring complete V1-V9 region. Using SMRT technology, we identified OTUs based on high-quality sequences processed through Mothur package against green gene_13_8_99.gg.tax database for species richness in 37 CP granulation tissue samples. Among these a total of 112 observational IDs were unclassified and 125 were classified at the species level (Supplementary Figures 2a and 2b; Supplementary Table 4). These unclassified species may represent microbial communities' specific for granulation tissues for which full-length OTU IDs have not been characterized in green gene database at the species level. Although some reports have used a different database for comprehensive taxonomic identification, the limitation in our study was that PICRUST utilizes green gene database based OTU IDs for functional classification. Thus, we cannot annotate these unclassified species.

Under normal conditions, microorganism reported in the healthy sub- and supragingival plaque showed a decrease of* Firmicutes* compared to other superficial oral regions like hard palate, buccal mucosa, keratinized gingiva, saliva, and tongue. Genera-wise* Streptococcus* showed dominance in the sub- and supragingival plaque with an abundance of* Veillonella*,* Prevotella*,* Neisseria*,* Fusobacterium*,* Actinomyces*,* Leptotricha*, and* Cornybacteria*. The above evidence suggests decrease of* Firmicutes* was observed as depth increases in healthy samples [18]. Significant high relative abundance of* Synergistete* phyla [19] and* Firmicutes phyla *has also been reported under CP conditions compared to healthy conditions by Galimans [20]. Additionally, Phyla* Proteobacteria* and* Bacterioidetes,* as well as genera, Prevotella, Porphyromonas, and Treponema, showed high abundance across all subgingiva of severe chronic diseased samples [21, 22]. Popova et al. [3] reviewed and suggested* Porphyromonas gingivalis*,* Treponema denticola*, and* Tannerella forsythia* form a consortium in the subgingival biofilm and main periodonto-pathogenic bacteria [3], while other predominant species in the disease process were* Aggregatibacter actinomycetemcomitans*,* Fusobacterium nucleatum*,* Prevotella intermedia*,* Campylobacter rectus*,* Peptostreptococcus migros, *and* Eikenella corrodens* [3].

In our study, all deep seated-CP granulation tissue samples showed the prominence of* Firmicutes, Proteobacteria, *and* Bacteroidetes *(Supplementary Figure 2a). Most of the genera and identified species in the above phylum were gram-negative anaerobes [Firmicutes*-Veillonella parvula *(45.2%) and* Veillonella* ID1004529 (54.67%);* Selenomonas* ID1030589 (21.39%), and* Selenomonas noxia* (40.62%); Proteobacteria-*Neisseria* ID1007399 sp. 35.2% and* Neisseria oralis* 62.61%; Bacteroidetes-Prevotella ID1000563 sp. (51.2%),* Prevotella intermedia* (20%) and* Prevotella nigrescens* (12.59%) and* Capnocytophaga ochracea* sp. (67.8%) and* Capnocytophaga* ID1001498 (31.9%) (Supplementary Figure 2b)] [19]. Although the species* Selenomonas *and* Veillonella species *in phyla-Firmicutes*; Neisseria species *in Phyla Proteobacteria*; Capnocytophaga *and* Prevotella species *in phyla* Bacteroidetes *showed equivalent abundance as earlier reported [18] and some of the species have been reported in children with dental caries [23] except Firmcutes-*Streptococcus* (ID1000186-86.9%,* agalactiae*-12%,) species were gram-positive (Supplementary Figure 2b). This corroborates the fact that gram-negative bacteria play important role in tissue loss in CP, most likely due to their production of various virulence factor including collagenase, protease, and lipopolysaccharide endotoxins [3]. Additionally, high dipeptidase activity has been reported in* Proteobacteria* and* Bacteroidetes*  [24] which may further cause tissue damage.

Random distribution of microbial species was observed in HPV+ and HPV- CP samples (Supplementary Figures 2a and 3b) with significant dominance of* Capnocytophaga ochracea* in HPV+ samples (Figure 1).* Capnocytophaga ochracea*, a capnophilic aerotolerant anaerobe, has been reported to ferment hexoses and disaccharides into their respective phosphoesters. During this process, phospho-enol-pyruvate is used as a source of energy to regulate phosphotransferase pathway (PTS-in our study difference in mean proportion for PTS pathway was 0.1-0.2%; p=0.035) (Figure 2), which transports sugar across bacterial cell membrane [25]. Also,* C. ochreace* have adhesion sites (*L. rhamnose* sensitive, N acetylneuraminic acid sensitive sites) which help it to bind to other bacterial species like* Streptococcus* and* Fusobacteria* [26]. This coadhesion of species contributes to early dental plaque formation and may play important role in causing periodontitis as suggested by Popova et. 2013 [3]. Dominance of* C. ochreace* in HPV+ sample may further explain the higher mean alveolar loss (3.90 mm) in HPV+ tongue SQCC-CP cases compared to 2.85 mm in HPV- tongue SQCC-CP cases (a retrospective study by Guerrero-Preston et al., 2016) [8]. On comparing HPV+ and HPV- cases, NER, mismatch repair pathway showed a significant decrease in HPV+ samples suggesting the higher formation of DNA adducts, which may lead to the process of carcinogenesis.

Membersof* Porphyromonas genera*,* Macellibacteroides fermentans* and* Porphyromonas endodontalis*,* Campylobacter rectus*, and* Treponema phagedenis* showed prominence in HPV- CP samples (Figure 1).* Porphyromonas* sp. and* Campylobacter* sp. are responsible for beta-lactamase and bone loss activities, respectively [2]. * Porphyromonas endodontalis*, gram negative black-pigmented anaerobes, and* Macellibacteroides fermentans* (obligate anaerobe) have also been shown to be associated with CP [2, 27]. Porphyromonas species do not ferment carbohydrates; instead, they use proteinaceous substrates as carbon and energy source and also possess dipeptidyl peptidase (DPP) 7 peptidase activity [8] which may lead to tissue loss in CP.* Campylobacter rectus* has also been reported as periodontal pathogens which invade into deeper tissue. It also shows commensalism with* Porphyromonas gingivalis* [3]. Both* Porphyromonas endodontalis* and* Campylobacter rectus* have been reported in orange complex and similar to the red complex have been shown to have a significant association with increasing pocket depth [19, 20].

Based on nonmetric multidimensional scaling plot (Supplementary Figure 6a) and heat map profile (Supplementary Figure 6b), we observed no species difference between male and female CP granulation tissue which corroborated with human oral microbiome study which showed no specific difference between female and males under normal conditions. However, through Picrust analysis, we identified* Pseudoxanthomonas kaohsiungensis* which showed prevalence in CP females (Figure 3) and* Desulfobulbus rhabditiform* (gram negative) in granulation tissue of male CP patients.* Desulfubulbus rhabdoformis* (gram negative) in granulation tissue of male CP patients which has been previously associated with periodontitis in the CP-smokers (Figure 3) [27, 28] and is also associated with poor response to periodontal therapy [27]. Also inorganic sulfate has been determined by controlled-flow anion chromatography in human saliva and sweat may be a substrate for* Desulfubulbus rhabdoformis*[24] and the main product of reduction is hydrogen sulphide which is potentially toxic.

Due to enhanced abundance of* Desulfubulbus rhabdoformis*, upregulation of Lipopolysaccharide biosynthesis (p=0.047), protein metabolism including alanine, aspartate, and glutamate metabolism (p=0.041), and arginine and proline metabolism as well as carbon fixation pathways (p=0.011) was observed both in our (Figure 4) and Bizzarro's studies [28].

Although another species,* Pseudoxanthomonas mexicana*, identified in below 40 years of age group (Figure 5) has been reported to possess dipeptidyl aminopeptidase [23], which may play important role in tissue loss in CP, we also identified upregulation of benzoate degradation, ascorbate and aldorate metabolism, bacterial toxins, valine, leucine, and isoleucine degradation, synthesis, and degradation of ketone bodies pathways in females and of secretion system and bacterial secretion system, pathways in below 40 years of age group (Figure 6).

Hence, we conclude that dominance of gram negative species in granulation tissue of severe CP cases with peptidase activity in deep seated granulation tissue of severe CP cases may be responsible for increased depth of periodontal pocket. They also release virulence factors which utilize the multitude of methods to invade mammalian host, damage tissue sites, and thwart the immune system. This has also been suggested through other studies which showed a link between proteobacteria and dysbiosis, to explain the potential relationship between inflammation and gene level differences in gut microbial functions [29]. Further, an association of HPV with granulation tissue-microbiota under anaerobic conditions enhances fermentation of hexoses using PEP (phosphoenolpyruvate) as a source of energy may synergize various processes and may create an environment favouring carcinogenesis. Hence, granulation tissue may serve as good predisposition marker for oral cancer.

## Figures and Tables

**Table 1:** Summary of the primer pair used to generate the 16S rDNA gene fragment fragments and the characteristics of each region in CP samples.

Region		Forward Primers (with forward barcode-Red)	Amplicon length (Without Barcode)
V1-V9	F	AGRGTTYGATYMTGGCTCAG	1,464
	F-1	TGAGTGACGTGTAGCGAGRGTTYGATYMTGGCTCAG	
	F-2	GACAGCATCTGCGCTCAGRGTTYGATYMTGGCTCAG	
	F-3	TGCGAGCGACTCTATCAGRGTTYGATYMTGGCTCAG	
	F-4	TGCTCTCGTGTACTGTAGRGTTYGATYMTGGCTCAG	

Region		Reverse Primers (with reverse barcode-Red)	

V1-V9	R	RGYTACCTTGTTACGACTT	1,464
	R-1	GCTCGACTGTGAGAGA RGYTACCTTGTTACGACTT	
	R-2	TGCTCGCAGTATCACA RGYTACCTTGTTACGACTT	
	R-3	GCAGACTCTCACACGC RGYTACCTTGTTACGACTT	
	R-4	AGACAGCATCTGCGCTCRGYTACCTTGTTACGACTT	

where

F= Forward primer,

F-1= Forward barcode primer 1,

F-2= Forward barcode primer 2,

F-3= Forward barcode primer 3,

F-4= Forward barcode primer 4

R= Reverse Primer,

R-1= Forward barcode primer 1,

R-2= Forward barcode primer 2,

R-3= Forward barcode primer 3,

4-4= Forward barcode primer 4,

R = A or G,

Y = C or T,

M = A or C, and

N = any base.

## Data Availability

The Sequence files and metadata for all samples used to support the findings of this study have been deposited in the NCBI repository (SRA Accession: SRR7088851, SRR7088868, SRR7088872, SRR7088845, SRR7088847, SRR7088852, SRR7088855, SRR7088842, SRR7088841, SRR7088844, SRR7088870, SRR7088859, SRR7088854, SRR7088863, SRR7088869, SRR7088856, SRR7088864, SRR7088866, SRR7088853, SRR7088857, SRR7088858, SRR7088867, SRR7088865, SRR7088860, SRR7088862, SRR7088861, SRR7088871, SRR7088846, SRR7088843, SRR7088849, SRR7088848 and SRR7088850 under Bio Project (PRJNA451246) and Biosample (SAMN08966100).

## Additional Points

*Originality Significance Statement.* The work carried out in this manuscript is original, describing Dysbacteriosis induced severe Chronic Periodontitis (CP) cases which have been correlated with human papiloma virus (HPV) causing oral cancer. Highly abundant* Capnocytophaga ochracea* in HPV+ CP samples compared to* Porphyromonas endodontalis*,* Macellibacteroides fermentas*, and* Treponema phagedenis* and* Campylobacter rectus* species in HPV- samples lead to downregulation of mismatch-repair and nucleotide excision-repair, with cytoskeleton-proteins creating an environment favoring carcinogenesis. The abundance of above species in HPV+ granulation-tissue may serve as good predisposition marker for oral-cancer.

## Supplementary Materials

Supplementary Materials Supplementary Table 1: Selection of CP patients and their status related to Human Papiloma Virus (HPV+/HPV-) used in present study. Supplementary Table 2: Multiplexing with PacBio Barcoded Universal Primers in CP samples. Supplementary Table 3: The number of processed full-length 16S rRNA sequences per CP samples ranged from 672 to 28,780 with an average of 7,357 reads/sample. Supplementary Table 4: Observed number of OTUs at a 3% dissimilarity level and the diversity estimates. Supplementary Table 5a: Alpha-Diversity values of Shannon index for individual CP samples. Supplementary Table 5b: Alpha-Diversity Analysis tests ANNOVA (Repeated Measures ANNOVA) equal means. Supplementary Table 5c: Tukey's pairwise test showing no significant difference between sampled species in each sample. Supplementary Table 6: Different indexes reporting Beta-Diversity between HPV+ and HPV- CP dataset. Supplementary Figure 1a: Rarefaction Curves calculated at 3% dissimilarity compare the number of reads with the number of operational taxonomic units (OTUs) found in the DNA from the 40 CP samples. The curves for individual samples which nearly plateaued off for the majority of the samples. Supplementary Figure 1b: Sample rarefaction (Mao tau) curve of Alpha-Diversity for species richness estimation (p<0.05) as a function of number of samples derived from Shannon index values of individual samples. Supplementary Figure 2a: Diversity of bacterial phyla identified in granulation tissue of CP samples. Supplementary Figure 2b: Diversity of bacterial species in CP samples identified in granulation tissue. Supplementary Figure 3a: Scatter plot and correlation estimates of bacterial species abundance between HPV+ and HPV- samples in CP samples. Supplementary Figure 3b: Beta-Diversity nonmetric multidimensional scaling plot based on Theta YC two axis coordinate values obtained through Mothur using Weighted UniFrac algorithm, showing distance or dissimilarity between each sample pair. Supplementary Figure 3c: Principal Coordinates Analysis (PCoA) plot from ThetaYC values of beta diversity distance matrix, where coordinate 1 (X-axis) shows the maximum amount of variation present in the samples, with respect to coordinate 2 (Y-axis). Representation of OTU's between HPV+ and HPV- samples is randomly distributed. Supplementary Figure 4: Heat-map profile of abundance distribution of identified species in CP samples. Supplementary Figure 5a: Scatter plot and correlation estimates of bacterial species functional predictions between HPV+ and HPV- samples in CP samples. Supplementary Figure 6a: Scatter plot and correlation estimates of bacterial species abundance between male and female samples in CP samples. Supplementary Figure 6b: Heat-map profile of abundance distribution of identified species in male and female samples in CP samples. Supplementary Figure 7a: Heat-map profile of functional predictions of identified species in male and female samples in CP samples. Supplementary Figure 7b: Mean proportion of bacterial species abundance between male and female CP samples. The significant differences observed between the two groups at 95% confidence level and p<0.05 are reported. Supplementary Figure 8a: Scatter plot and correlation estimates of bacterial species abundance between above and below 40 years of age samples in CP samples. Supplementary Figure 8b: Heat-map profile of abundance distribution of identified species in above and below 40 years of age samples in CP samples. Supplementary Figure 9a: Heat-map profile of functional predictions of identified species in above and below 40 years of age samples in CP samples. Supplementary Figure 9b: Mean proportion of bacterial species abundance between above and below 40 years of age samples in CP samples. The significant differences observed between the two groups at 95% confidence level and p<0.05 are reported.
