# Supplementary material for: Dysbiosis and Variation in Predicted Functions of the Granulation Tissue Microbiome in HPV Positive and Negative Severe Chronic Periodontitis
Source: Biomed Res Int. 2019 Apr 14;2019:8163591. doi: 10.1155/2019/8163591 (PMC6487177; doi:10.1155/2019/8163591)
Supplement: Supplementary Materials — Supplementary Table 1: Selection of CP patients and their status related to Human Papiloma Virus (HPV+/HPV-) used in present study. Supplementary Table 2: Multiplexing with PacBio Barcoded Universal Primers in CP samples. Supplementary Table 3: The number of processed full-length 16S rRNA sequences per CP samples ranged from 672 to 28,780 with an average of 7,357 reads/sample. Supplementary Table 4: Observed number of OTUs at a 3% dissimilarity level and the diversity estimates. Supplementary Table 5a: Alpha-Diversity values of Shannon index for individual CP samples. Supplementary Table 5b: Alpha-Diversity Analysis tests ANNOVA (Repeated Measures ANNOVA) equal means. Supplementary Table 5c: Tukey's pairwise test showing no significant difference between sampled species in each sample. Supplementary Table 6: Different indexes reporting Beta-Diversity between HPV+ and HPV- CP dataset. Supplementary Figure 1a: Rarefaction Curves calculated at 3% dissimilarity compare the number of reads with the number of operational taxonomic units (OTUs) found in the DNA from the 40 CP samples. The curves for individual samples which nearly plateaued off for the majority of the samples. Supplementary Figure 1b: Sample rarefaction (Mao tau) curve of Alpha-Diversity for species richness estimation (p<0.05) as a function of number of samples derived from Shannon index values of individual samples. Supplementary Figure 2a: Diversity of bacterial phyla identified in granulation tissue of CP samples. Supplementary Figure 2b: Diversity of bacterial species in CP samples identified in granulation tissue. Supplementary Figure 3a: Scatter plot and correlation estimates of bacterial species abundance between HPV+ and HPV- samples in CP samples. Supplementary Figure 3b: Beta-Diversity nonmetric multidimensional scaling plot based on Theta YC two axis coordinate values obtained through Mothur using Weighted UniFrac algorithm, showing distance or dissimilarity between each sample pair. Supplement [file 8163591.f1.pdf]

Supplementary Table 1: Selection of CP Patients and their status related to Human Papiloma Virus (HPV+/HPV-) used in present study.

	Age	Sex	HPV status
CP1	26	F	positive
CP2	22	M	negative
CP3	30	F	negative
CP4	22	F	negative
CP5	26	F	positive
CP6	50	F	negative
CP7	60	M	negative
CP8	60	F	negative
CP9	50	M	negative
CP10	37	M	negative
CP11	39	M	negative
CP12	34	M	negative
CP13	39	M	negative
CP14	22	F	negative
CP15	55	F	positive
CP16	44	F	negative
CP17	37	M	negative
CP18	40	F	negative
CP19	32	F	positive
CP20	66	F	negative
CP21	33	F	positive
CP22	18	F	negative
CP23	40	F	negative
CP24	39	F	positive
CP25	38	F	negative
CP26	48	M	positive
CP27	40	F	negative
CP28	30	M	negative
CP29	48	F	negative
CP30	36	M	negative
CP31	42	M	negative
CP32	32	M	negative
CP33	31	M	negative
CP34	30	F	negative
CP35	27	M	negative
CP36	42	M	negative
CP37	29	M	negative

CP38	49	M	negative
CP39	30	F	negative
CP40	35	F	negative

Supplementary Table 2: Multiplexing with PacBio Barcoded Universal Primers in CP samples

S. No	Sample Name	Barcoded Primers Pair Used (Forward and Reverse Primers)	Pooling of Barcoded Samples and Library preparation
1	CP-1	F-R	Library 1
2	CP-2	F1-R1	Library 2
3	CP-3	F2-R1	Library 3
4	CP-4	F2-R2	
5	CP-6	F2-R3	
6	CP-7	F2-R4	
7	CP-8	F3-R1	Library 4
8	CP-9	F3-R2	
9	CP-10	F3-R3	
10	CP-11	F3-R4	
11	CP-13	F4-R1	Library 5
12	CP-14	F4-R2	
13	CP-15	F4-R3	
14	CP-16	F4-R4	
15	CP-18	F1-R1	Library 6
16	CP-12	F3-R4	
17	CP-5	F4-R3	
18	CP-19	F1-R2	
19	CP-20	F1-R3	
20	CP-17	F1-R3	Library 7
21	CP-21	F1-R1	
22	CP-22	F2-R1	
23	CP-23	F3-R1	
24	CP-24	F4-R1	
25	CP-25	F1-R2	
26	CP-26	F2-R3	
27	CP-27	F3-R3	
28	CP-28	F1-R1	Library 8
29	CP-29	F1-R2	
30	CP-30	F1-R3	
31	CP-31	F1-R4	
32	CP-32	F2-R1	
33	CP-33	F2-R2	
34	CP-34	F2-R3	

35	CP-35	F2-R4	
36	CP-36	F3-R1	
37	CP-37	F3-R2	
38	CP-38	F3-R3	
39	CP-39	F3-R4	
40	CP-40	F4-R1	

Supplementary Table 3: The number of processed full-length 16S rRNA sequences per CP samples ranged from 672 to 28,780 with an average of 7,357 reads/sample.

Sample Name	Number of Raw CCS Reads	Number of CCS Reads after filtering
CP1	9475	4192
CP2	6556	3736
CP3	16309	10405
CP4	9724	5731
CP5	37158	28780
CP6	12727	7802
CP7	18378	11864
CP8	7825	6007
CP9	7613	5792
CP10	11478	8858
CP11	11737	8586
CP12	25108	20363
CP13	15397	13282
CP14	8138	6149
CP15	10887	8885
CP16	11549	9311
CP17	10286	7811
CP18	6707	5403
CP19	9406	7515
CP20	5860	4690
CP21	14434	11687
CP22	6391	4693
CP23	9393	7172
CP24	11301	8993
CP26	7533	5573

CP27	9707	7193
CP30	3457	3148
CP31	772	672
CP32	5528	5172
CP33	1913	1652
CP34	4241	3544
CP35	6622	4767
CP36	6391	5701
CP37	3306	2928
CP38	4925	4399
CP39	4941	4119
CP40	6123	5646
Total Reads	359296	272774
Average No of Reads	9710.70	7357.32
±SD	6656.78	5117.64

Supplementary Table 5a: Alpha diversity values of shannon index for individual CP samples

Group	Shannon	Shannon_Ici	Shannon_hci
CP1	6.866257	6.810809	6.921705
CP10	7.614023	7.578461	7.649585
CP11	7.292934	7.252411	7.333456
CP12	7.996757	7.967874	8.025639
CP13	7.557078	7.498929	7.615228
CP14	7.246837	7.194382	7.299292
CP15	7.708749	7.671141	7.746356
CP16	7.778777	7.738416	7.819138
CP17	7.486967	7.44582	7.528114
CP18	7.244874	7.204657	7.285091
CP19	7.604914	7.572032	7.637797
CP2	6.740064	6.692445	6.787683
CP20	7.077464	7.037181	7.117747
CP21	7.510457	7.456783	7.564131
CP22	7.005247	6.953505	7.056988
CP23	7.217533	7.17357	7.261496
CP24	7.600276	7.5583	7.642252
CP26	7.079197	7.027381	7.131013
CP27	7.38128	7.331073	7.431487
CP3	7.707934	7.666318	7.74955
CP30	6.8497	6.801366	6.898034
CP31	5.60211	5.517967	5.686252
CP32	6.105019	5.995159	6.21488
CP33	5.974474	5.901364	6.047585
CP34	6.665454	6.604803	6.726104
CP35	5.514053	5.420546	5.607561
CP36	6.901879	6.852621	6.951137
CP 37	6.587557	6.534557	6.640557
CP38	6.967821	6.921932	7.013711
CP39	6.992089	6.942125	7.042052
CP4	7.102627	7.047715	7.157538
CP40	6.785265	6.728457	6.842072
CP5	8.058471	8.017507	8.099434
CP6	7.799119	7.764647	7.833591
CP7	7.628564	7.596682	7.660446
CP8	7.111301	7.054793	7.16781
CP9	6.787735	6.730904	6.844566

Supplementary Table 5b: Alpha diversity analysis tests ANNOVA (Repeated Measures ANNOVA) equal means

	Sum of sqrs	df	Mean square	F	p (same)
Between groups	$7.19713E^{09}$	5	$1.43943E^{09}$	0.8979	**0.4837**
Within groups	-	222	$1.59909E^{09}$	-	-
Error	-	185	$1.60316E^{09}$	-	-
Between subjects	-	37	$1.57878E^{09}$	-	-
Total	-	227	-	-	-

Supplementary Table 5c: Tukey's pairwise test showing no significant difference between sampled species in each sample.

**nseqs**	**coverage**	**sobs**	**invsimpson**	**invsimpson_lci**	**invsimpson_hci**
nseqs	0.9999	1	0.7584	0.999	0.8509
coverage	0.3461	0.9999	0.6038	0.9892	0.7176
sobs	0.04313	0.303	0.7404	0.9985	0.8365
invsimpson	1.906	2.252	1.949	0.9286	1
invsimpson_hci	0.5389	0.885	0.582	1.367	0.9694
invsimpson_hci	1.656	2.002	1.699	0.2498	1.117

Supplementary Table 6: Different Indexes reporting Beta Diversity between HPV+ and HPV- CP dataset.

Whittaker:	2.1392
Harrison:	0.30561
Cody:	36.5
Routledge:	0.32126
Wilson-Shmida	3.6962
Mourelle:	0.52803
Harrison 2:	0.10317
Williams:	0.41935

Supplementary Figure 1a: Rarefaction Curves calculated at 3% dissimilarity, compare the number of reads with the number of operational taxonomic units (OTUs) found in the DNA from the 40 CP samples. The curves for individual samples which nearly plateaued off for the majority of the samples.

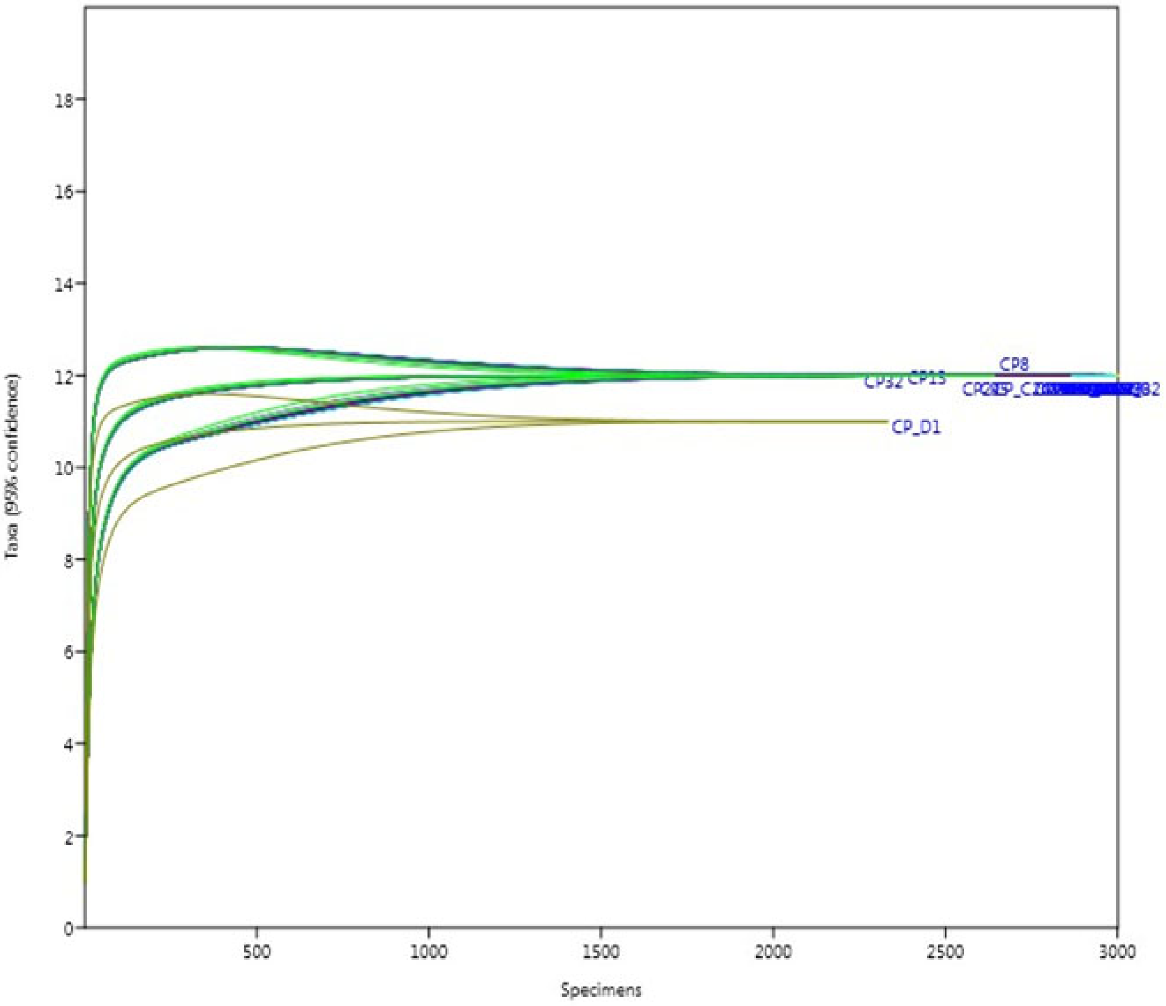

Supplementary Figure 1b: Sample rarefaction (Mao tau) curve of alpha diversity for species richness estimation ($p<0.05$) as a function of number of samples derived from Shanon index values of individual samples.

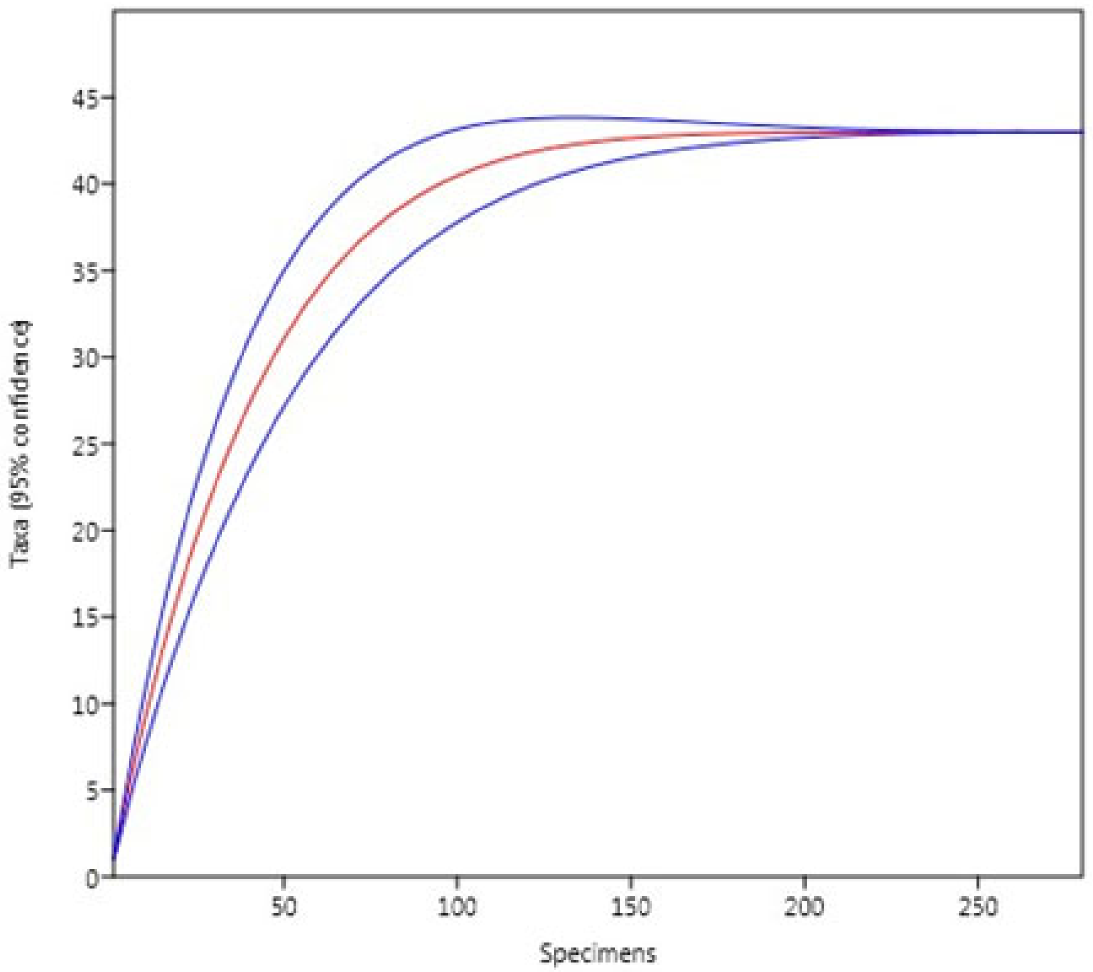

Supplementary Figure 2a: Diversity of bacterial phyla identified in granulations tissue of CP samples.

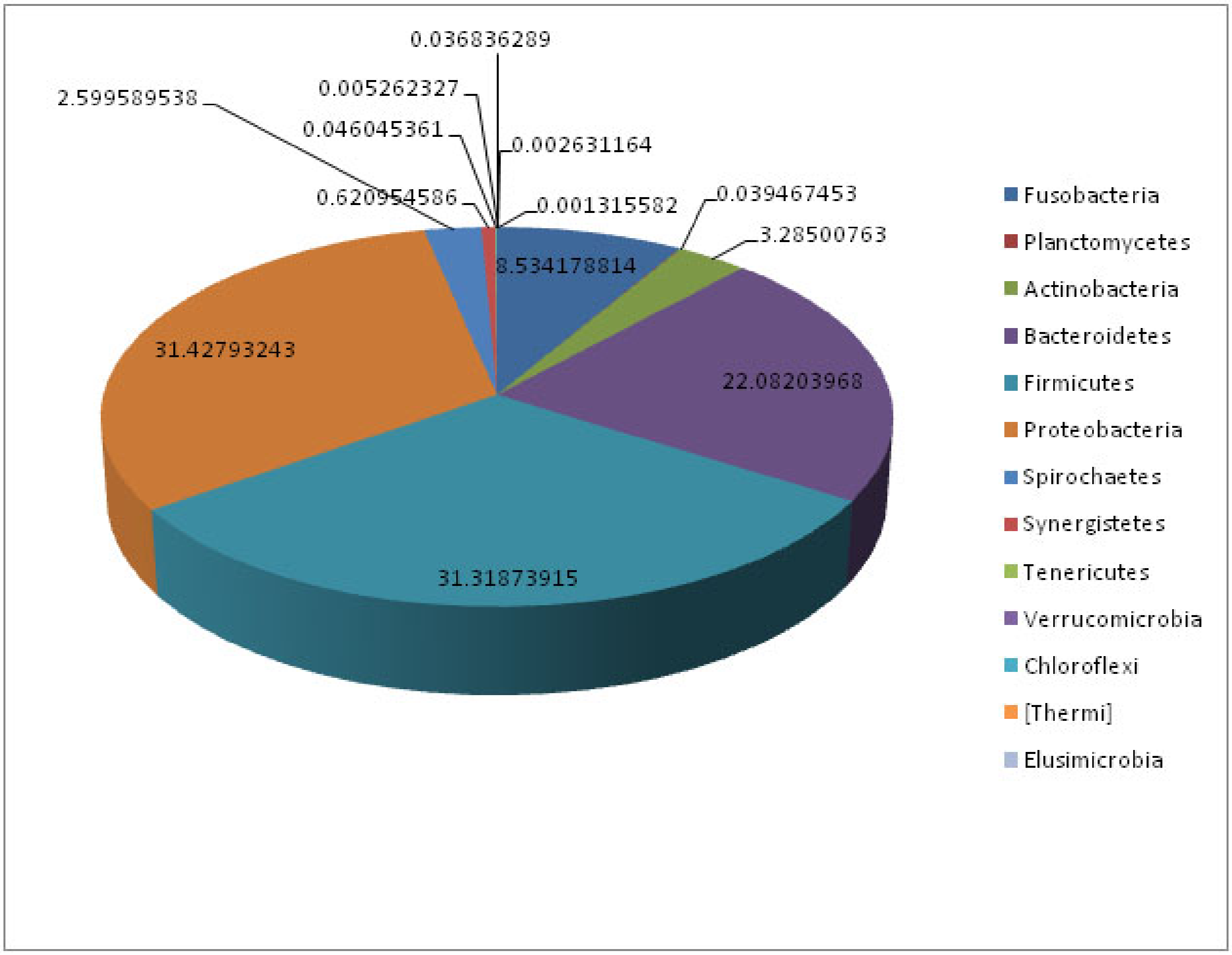

Supplementary Figure 2b: Diversity of bacterial species in CP samples identified in granulation tissue.

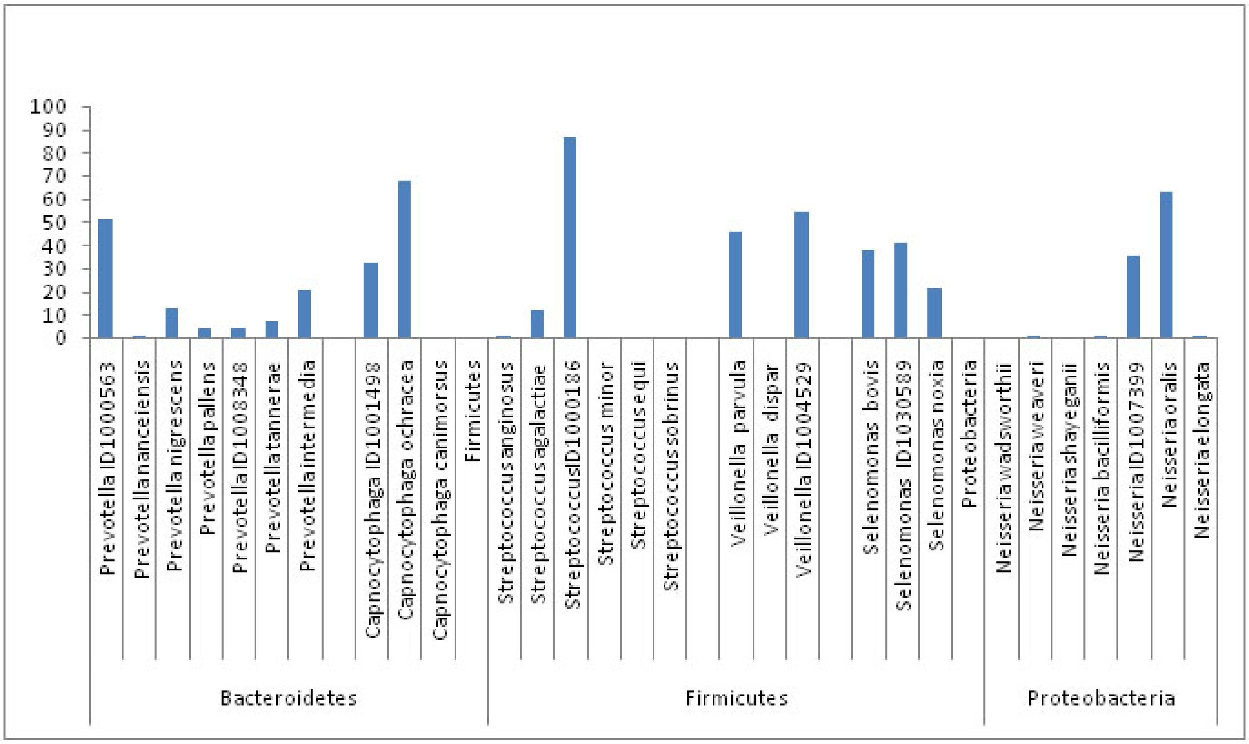

Supplementary Figure 3a: Scatter plot and correlation estimates of bacterial species abundance between HPV+ and HPV- samples in CP samples.

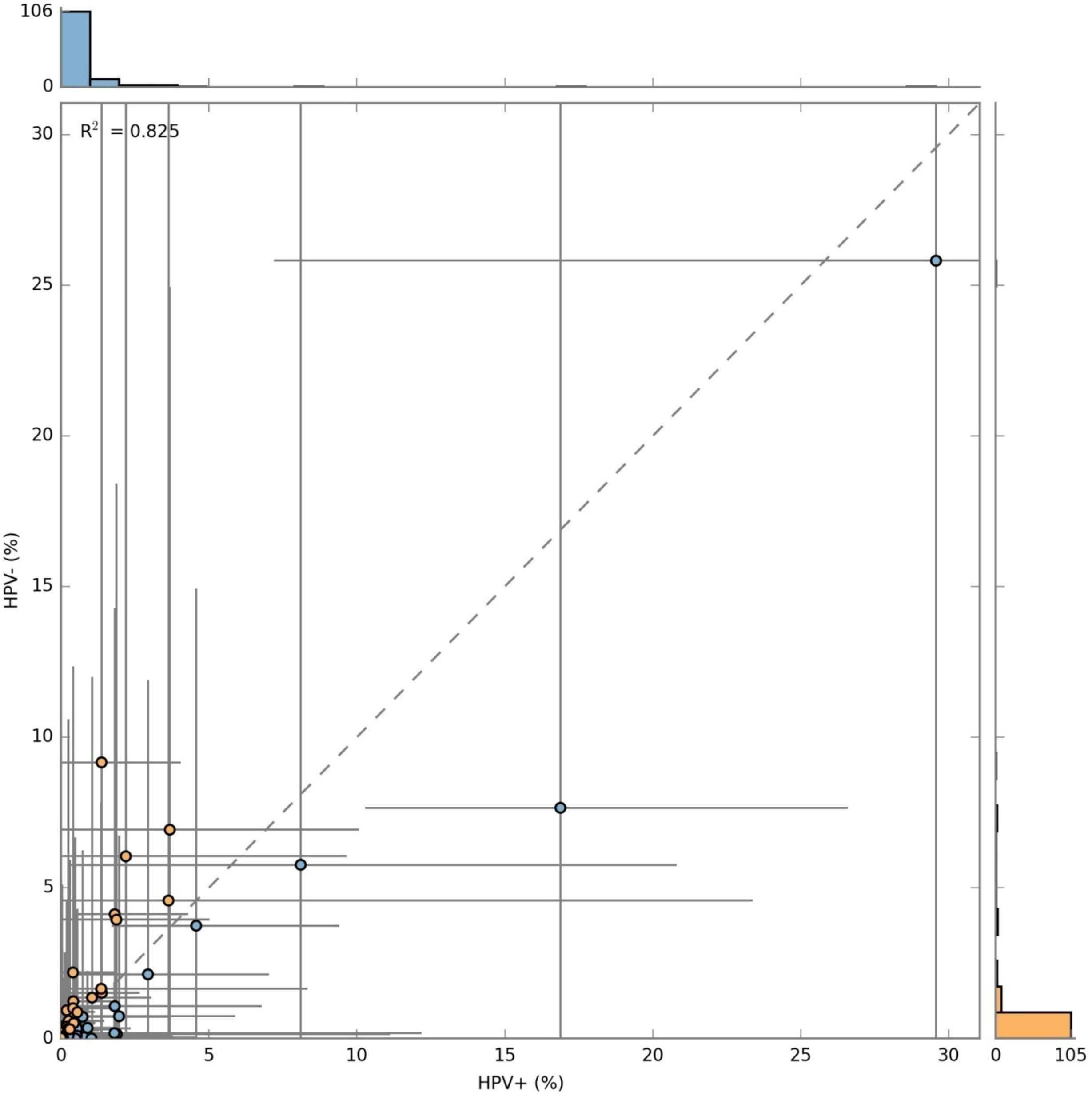

Supplementary Figure 3b: Beta diversity non-metric multidimensional scaling plot based on Theta YC two axis coordinate values obtained through mothur using Weighted UniFrac algorithm, showing distance or dissimilarity between each sample pair.

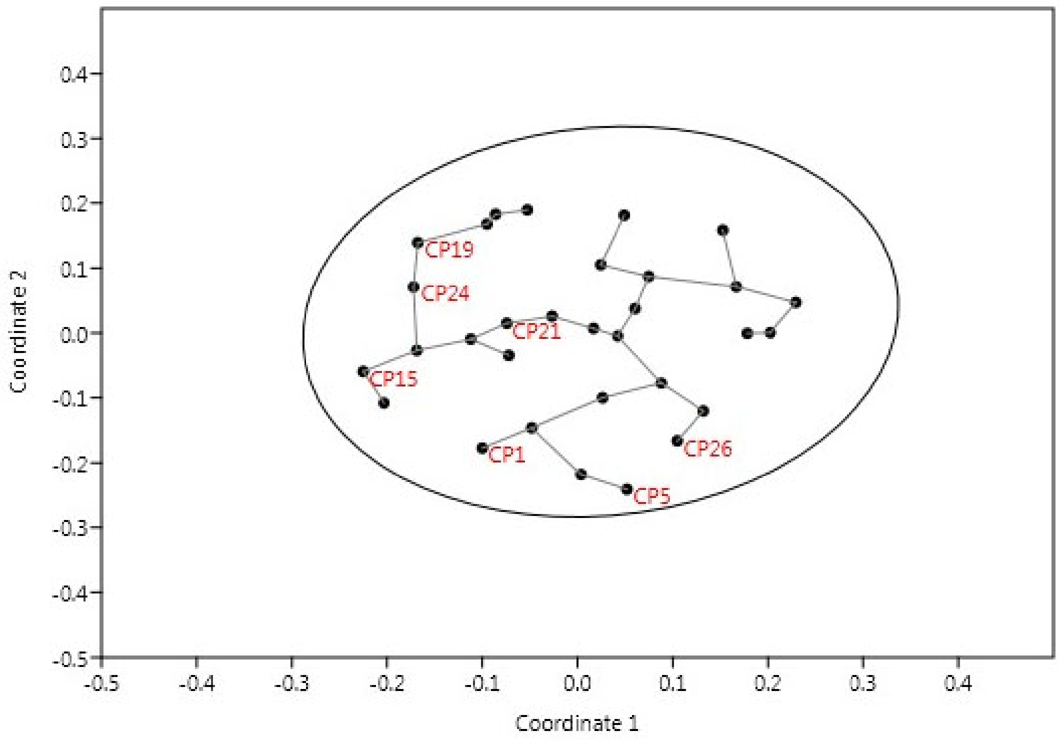

Supplementary Figure 3c: Principal coordinates analysis (PCoA) plot from ThetaYC values of beta diversity distance matrix. Where, coordinate 1 (X-axis) shows the maximum amount of variation present in the samples, with respect to coordinate 2 (Y-axis). Representation of OTU's between HPV+ and HPV - samples is randomly distributed.

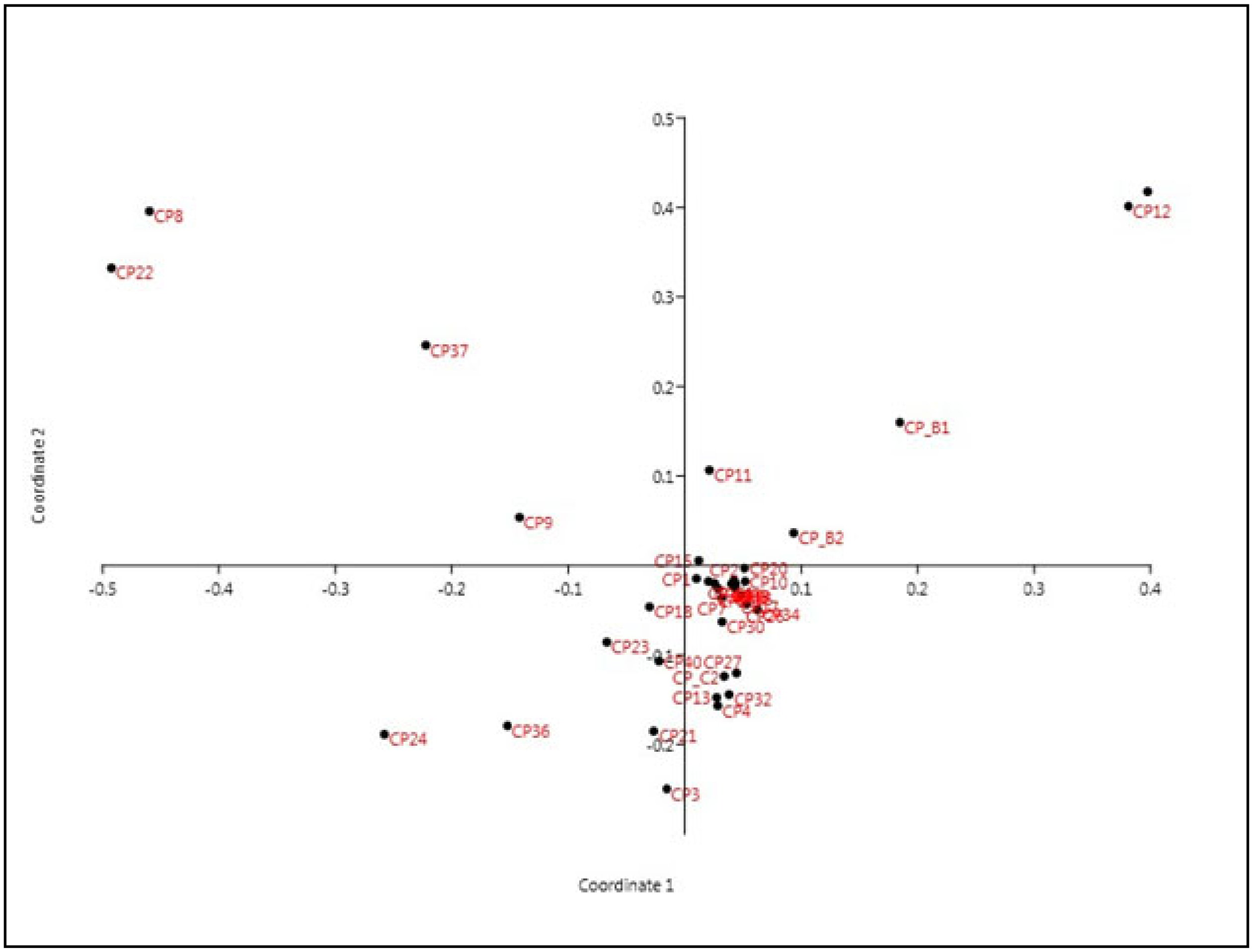

Supplementary Figure 4: Heat-map profile of abundance distribution of identified species in CP samples.

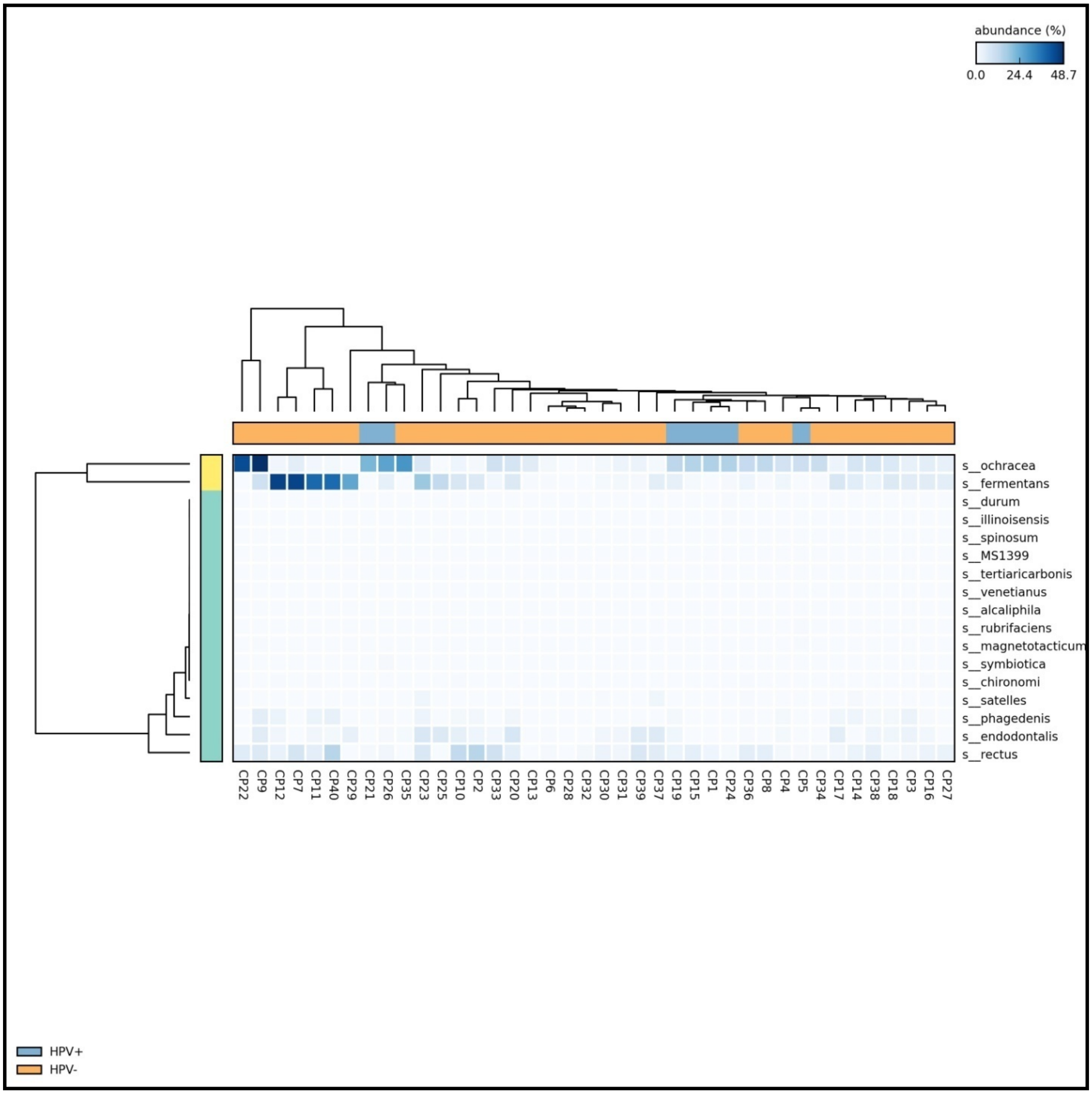

Supplementary Figure 5a: Scatter plot and correlation estimates of bacterial species functional predictions between HPV+ and HPV- samples in CP samples.

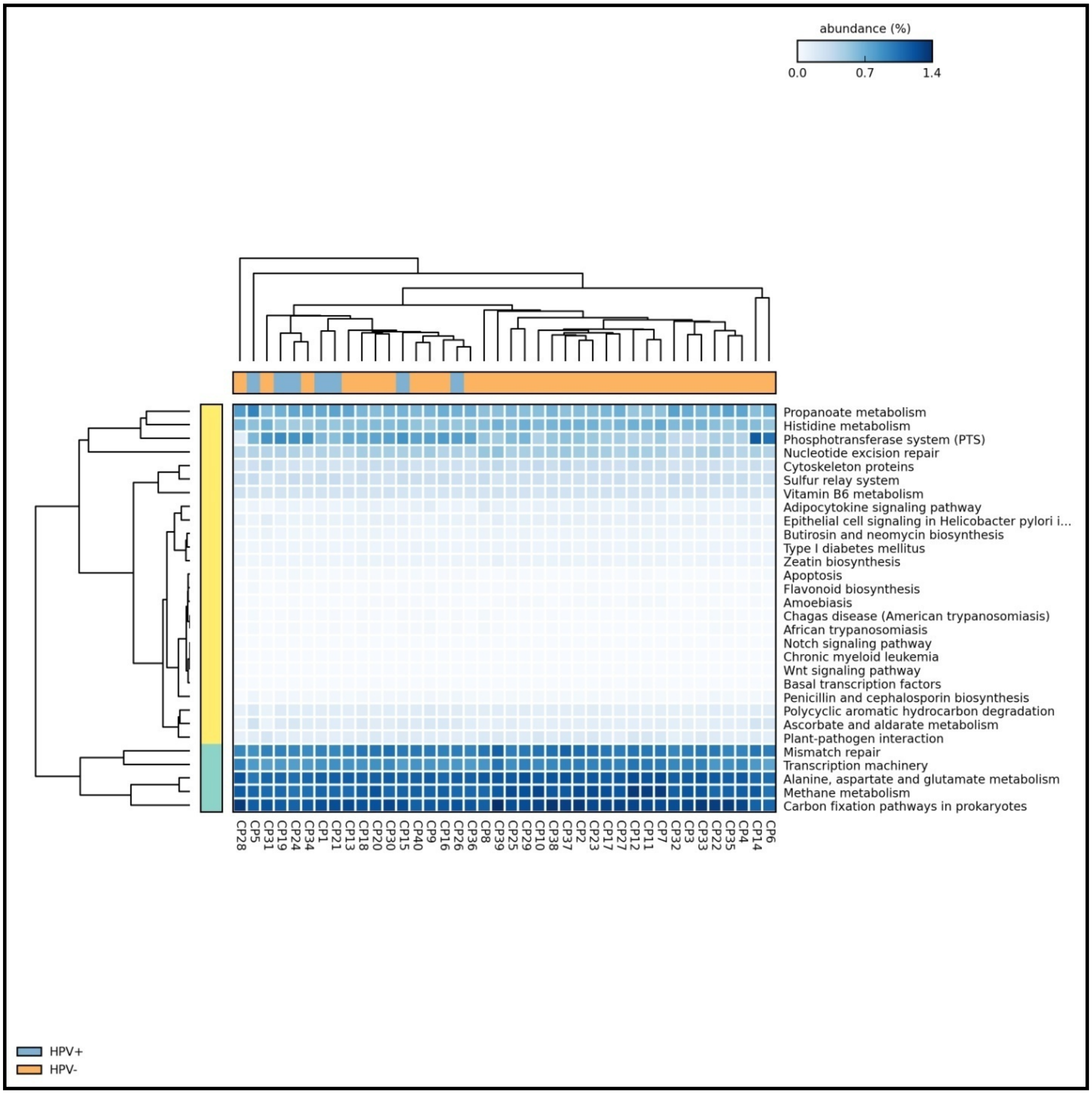

Supplementary Figure 6a: Scatter plot and correlation estimates of bacterial species abundance between Male and Female samples in CP samples.

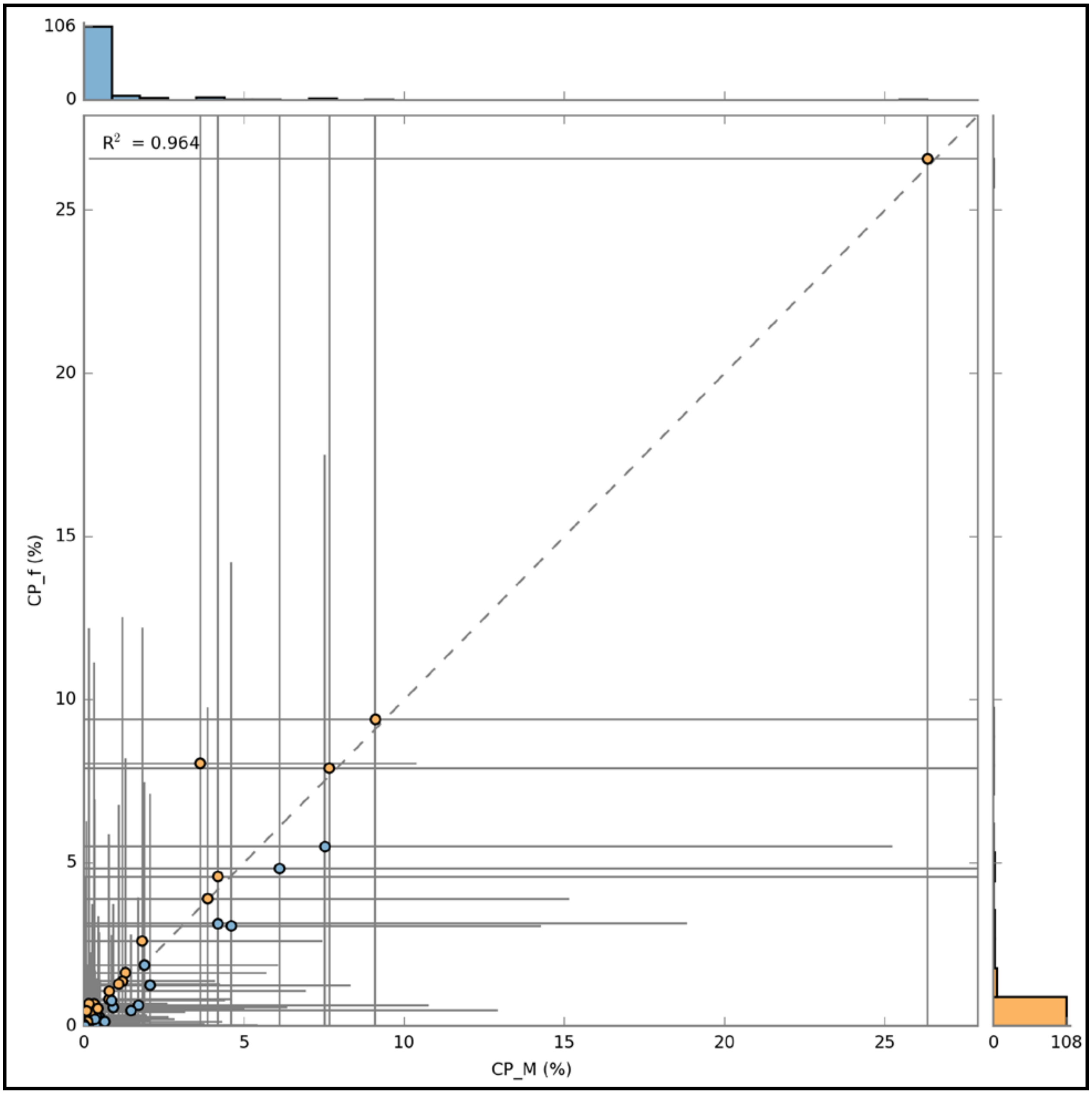

Supplementary Figure 6b: Heat-map profile of abundance distribution of identified species in Male and Female samples in CP samples.

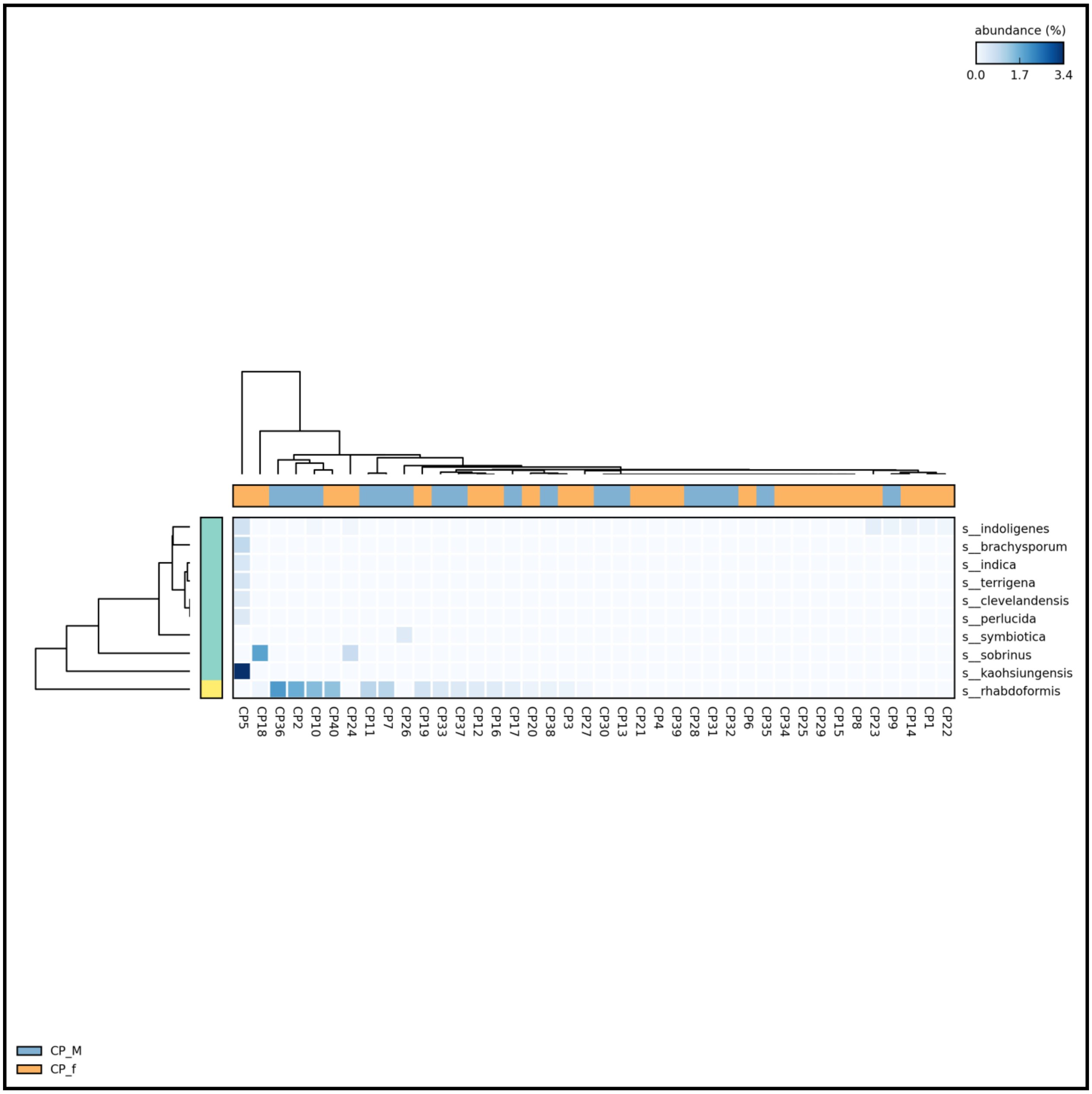

Supplementary Figure 7a: Heat-map profile of functional predictions of identified species in Male and Female samples in CP samples.

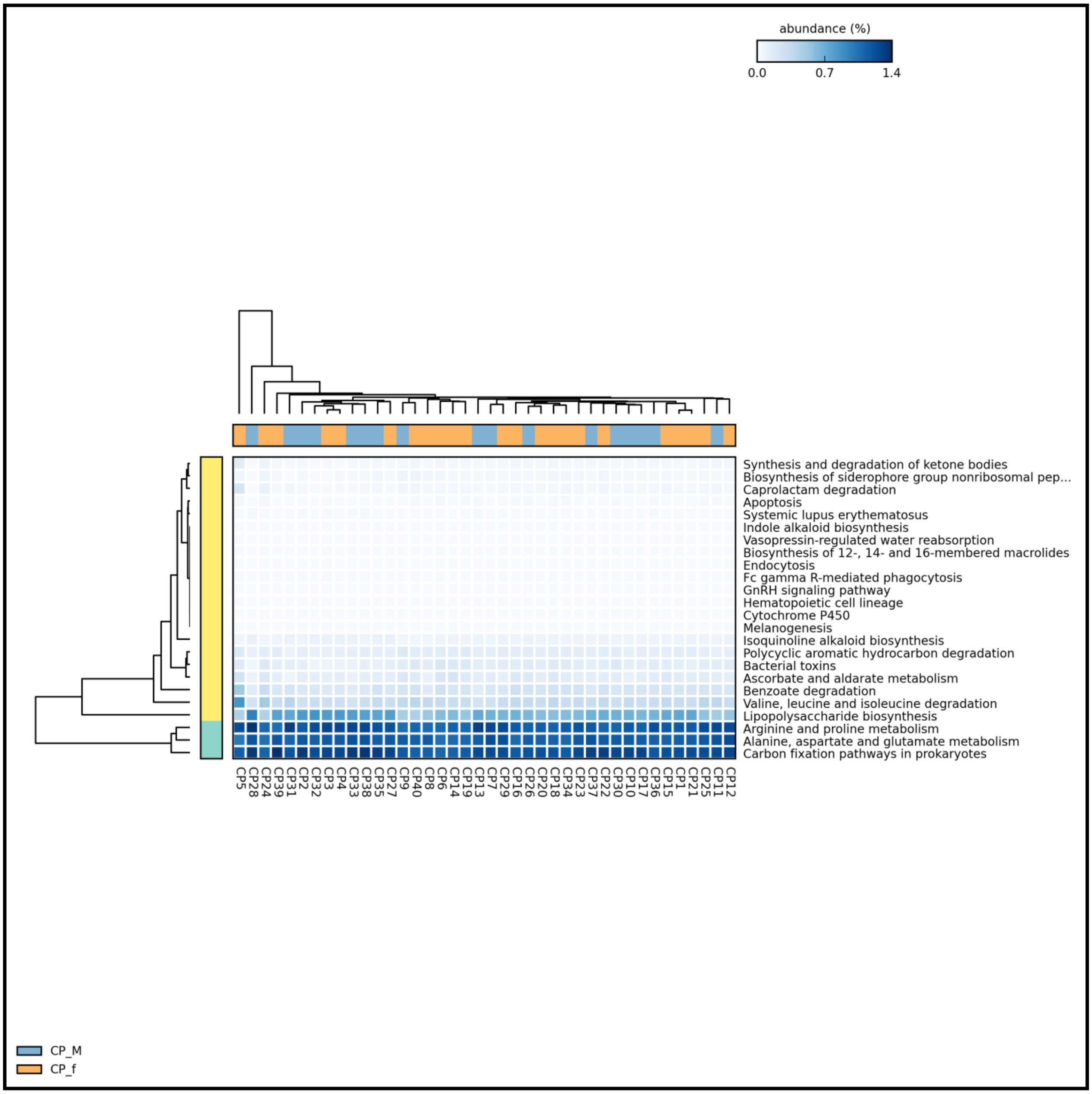

Supplementary Figure 7b: Mean proportion of bacterial species abundance between male and female CP samples. The significant difference observed between the two groups at 95% confidence level and p<0.05 are reported.

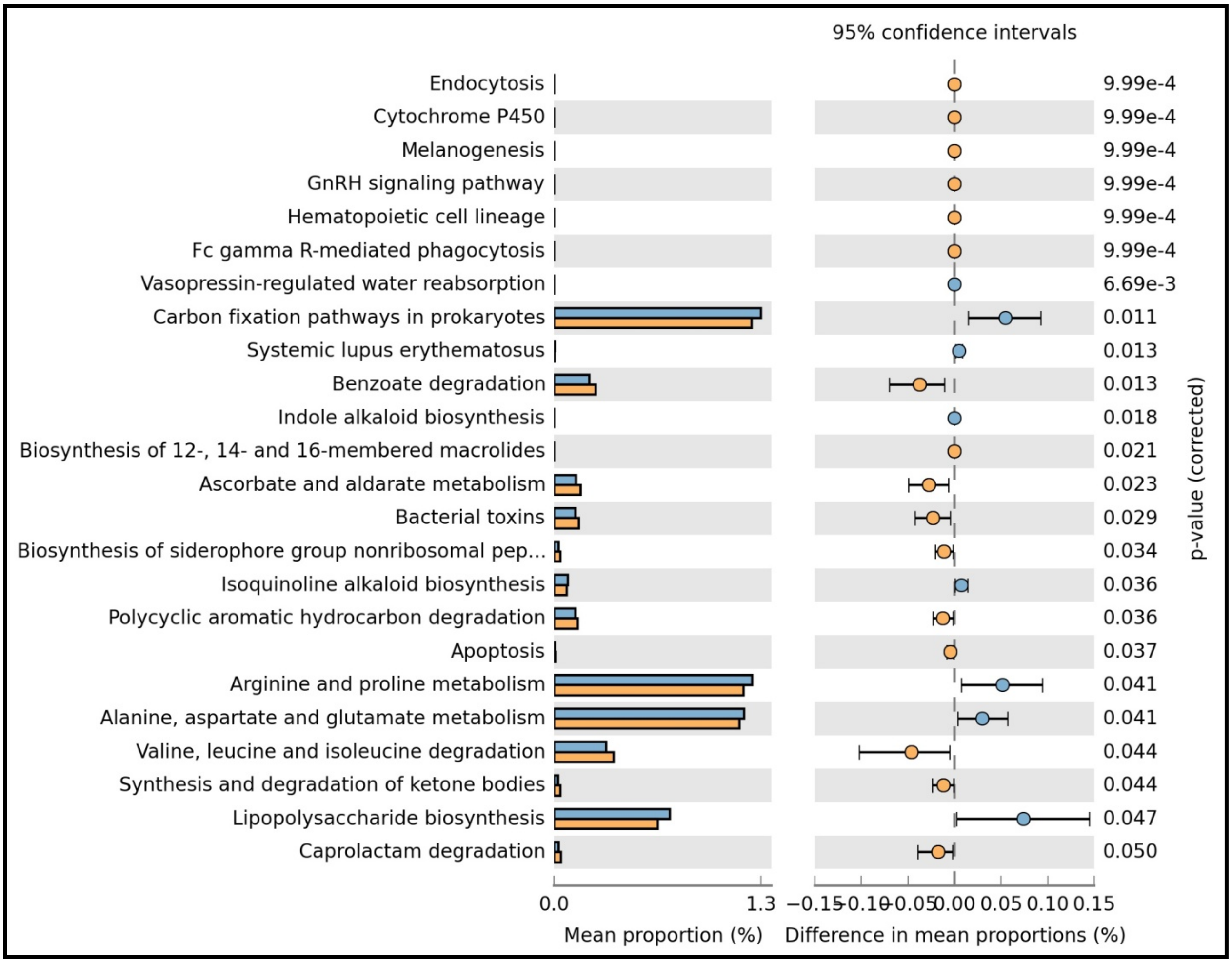

Supplementary Figure 8a: Scatter plot and correlation estimates of bacterial species abundance between Age above and below 40 samples in CP samples.

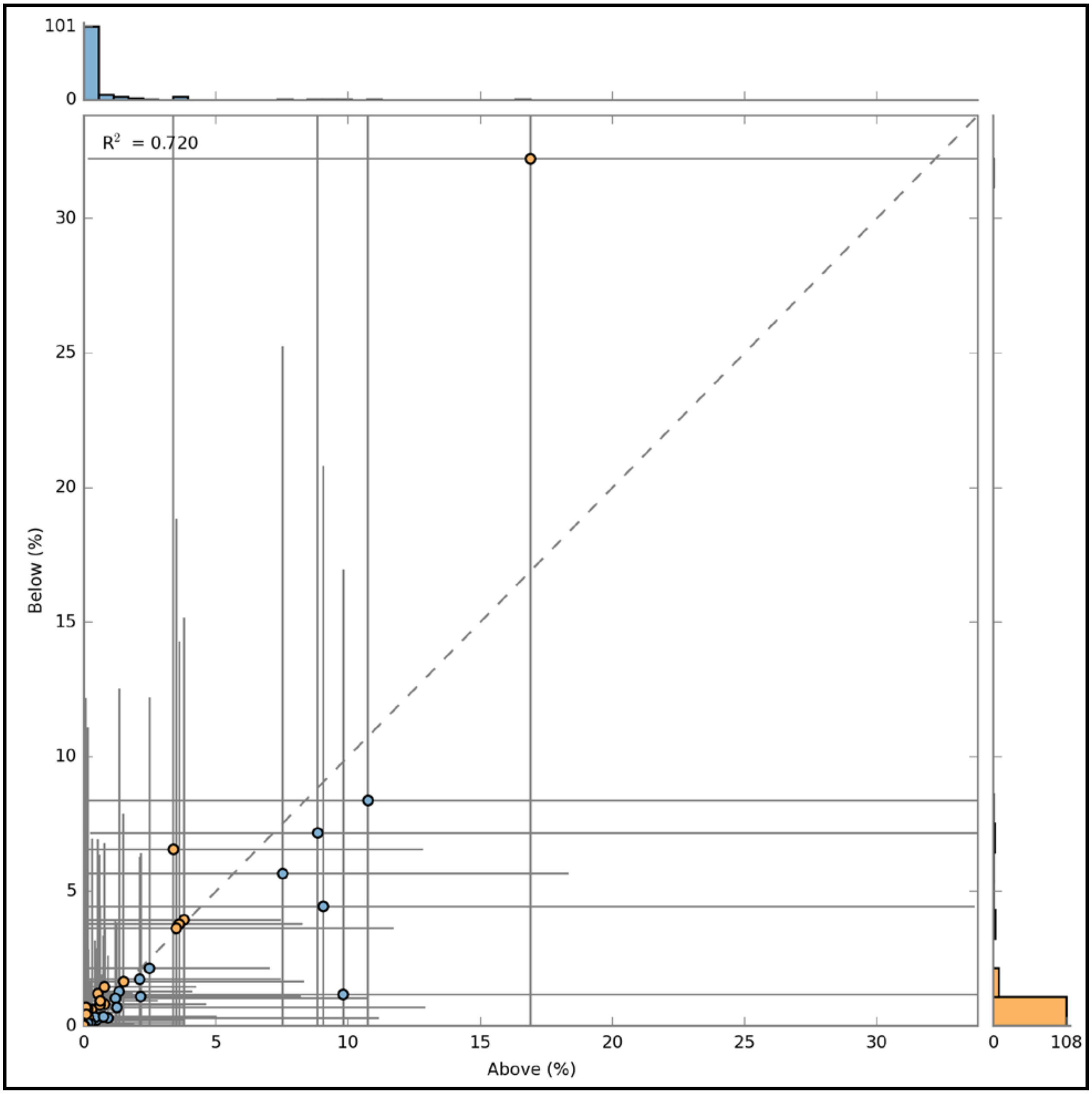

Supplementary Figure 8b: Heat-map profile of abundance distribution of identified species in Age above and below 40 samples in CP samples.

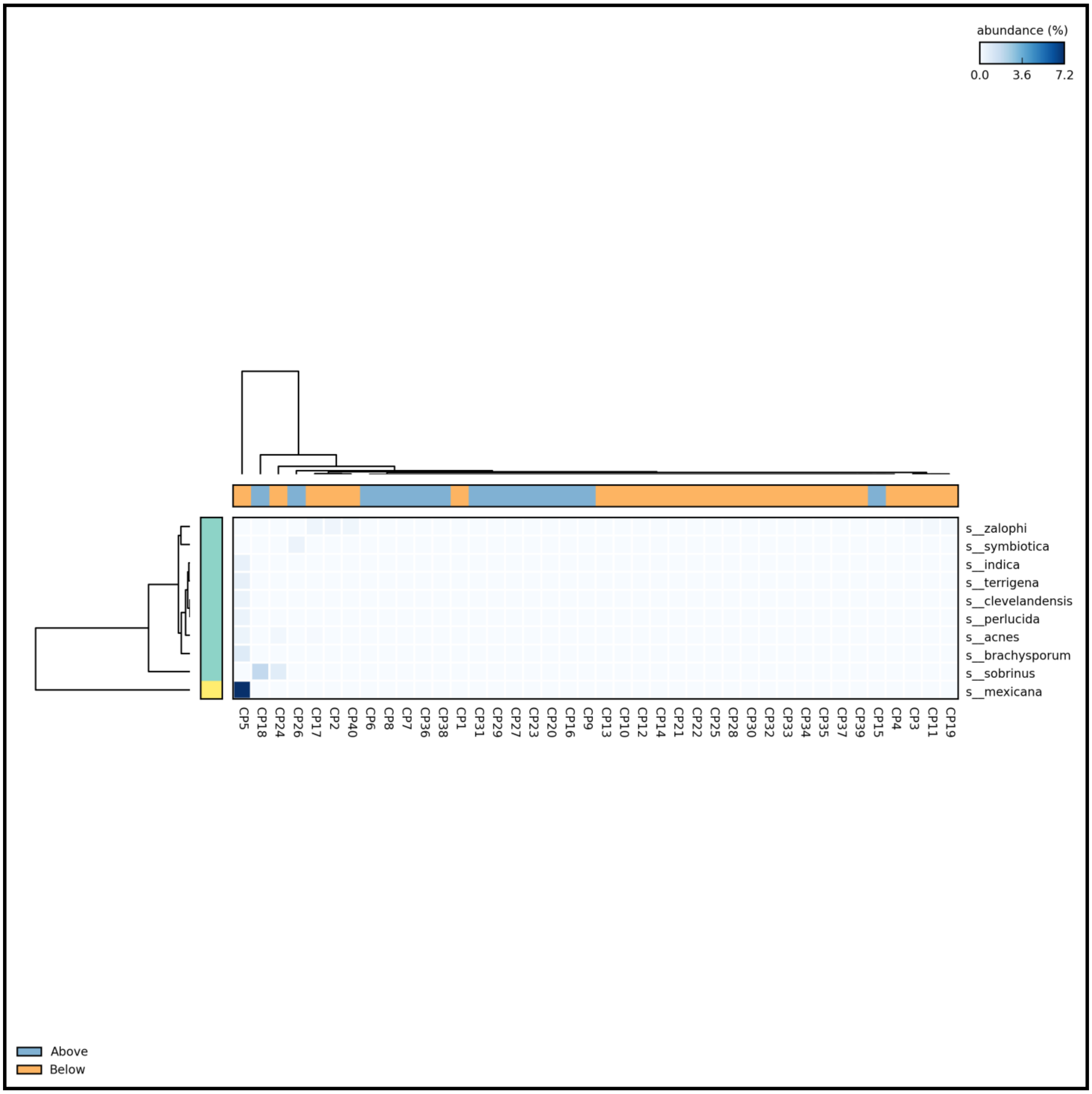

Supplementary Figure 9a: Heat-map profile of functional predictions of identified species in age above 40 (blue) and below 40 years (yellow) samples in CP samples.

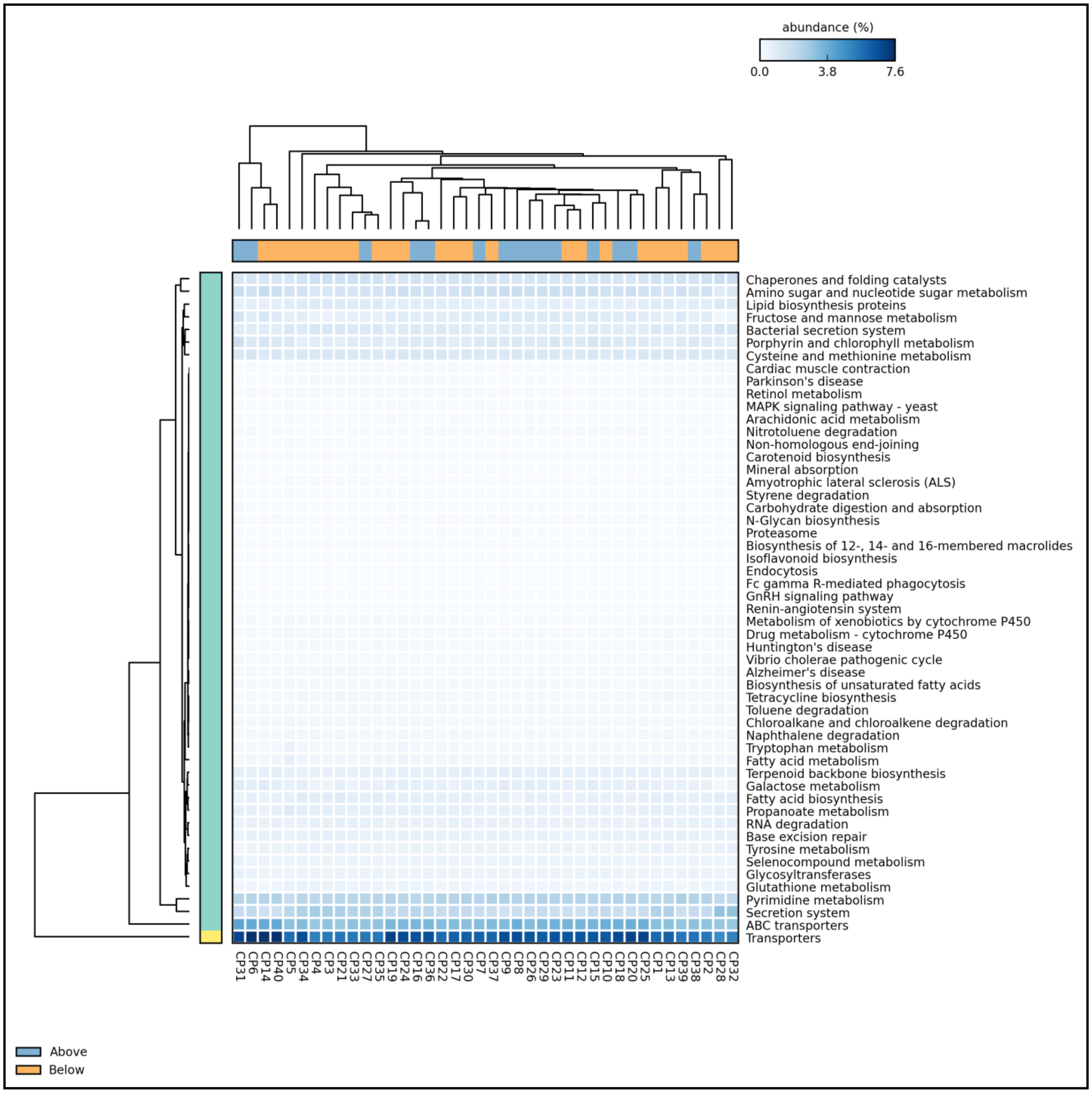

Supplementary Figure 9b: Mean proportion of bacterial species abundance between age above 40 (blue bar) and below 40 years (yellow bar) samples in CP samples. The significant difference observed between the two groups at 95% confidence level and p<0.05 are reported.

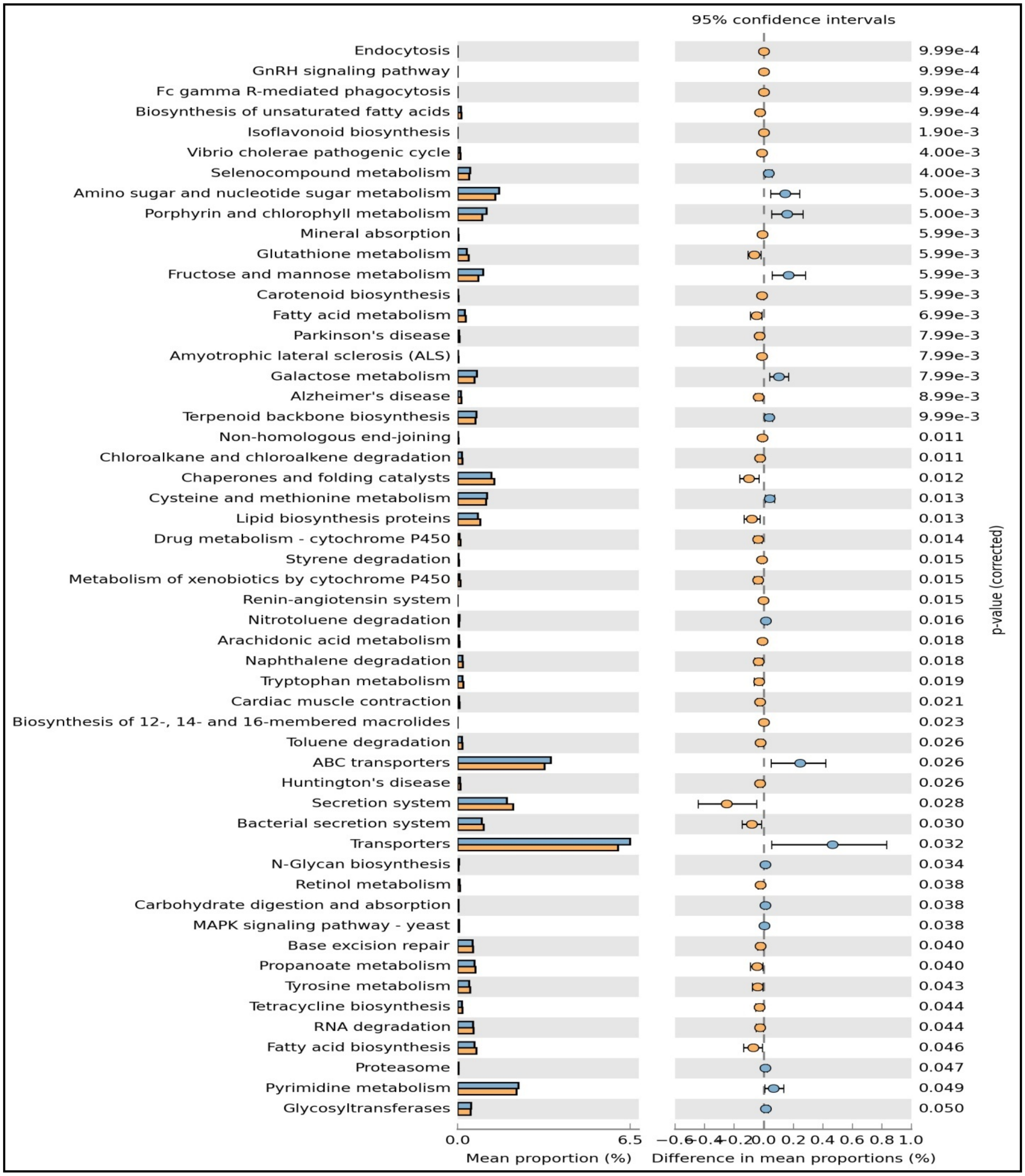

Supplementary Table 4: Observed number of OTUs at a 3% dissimilarity level and the diversity estima database for species richness in forty CP samples.

Level_1	Level_2	Level_3	Level_4	Level_5	Level_6	Level_7	Observatio	CP1
k__Bacteri	p__Actinob	c__Actinob	o__Actinor	f__Propion	g__Propior	s__acnes	ID1000517	0
k__Bacteri	p__Bacterc	c__Bactero	o__Bacterc	f__Prevote	g__Prevote	s__nanceie	ID1001920	1
k__Bacteri	p__Firmicu	c__Clostrid	o__Clostric	f__Lachnos	g__Lachno	s__orale	ID1009036	1
k__Bacteri	p__Firmicu	c__Clostrid	o__Clostric	f__Veillone	g__Veillone	s__parvula	ID1009968	37
k__Bacteri	p__Proteob	c__Gamma	o__Pseudo	f__Moraxe	g__Acineto	s__schindle	ID1012848	0
k__Bacteri	p__Proteob	c__Gamma	o__Pasteur	f__Pasteur	g__Haemo	s__parainfl	ID1016422	1
k__Bacteri	p__Actinob	c__Actinob	o__Actinor	f__Coryneb	g__Corynel	s__durum	ID1017764	0
k__Bacteri	p__Proteob	c__Gamma	o__Pasteur	f__Pasteur	g__Actinob	s__parahae	ID1019193	0
k__Bacteri	p__Firmicu	c__Clostrid	o__Clostric	f__Veillone	g__Veillone	s__dispar	ID1019878	0
k__Bacteri	p__Firmicu	c__Bacilli	o__Lactoba	f__Lactoba	g__Lactoba	s__salivariu	ID1021172	0
k__Bacteri	p__Actinob	c__Actinob	o__Actinor	f__Nocardi	g__Rhodoc	s__fascians	ID1021308	0
k__Bacteri	p__Bacterc	c__Bactero	o__Bacterc	f__Prevote	g__Prevote	s__nigresce	ID1028549	6
k__Bacteri	p__Bacterc	c__Flavoba	o__Flavoba	f__Flavoba	g__Capnoc	s__ochrace	ID102901	109
k__Bacteri	p__Proteob	c__Alphapr	o__Sphingo	f__Sphingo	g__Sphingo	s__asaccha	ID102915	0
k__Bacteri	p__Firmicu	c__Bacilli	o__Lactoba	f__Lactoba	g__Lactoba	s__iners	ID1033552	0
k__Bacteri	p__Bacterc	c__Bactero	o__Bacterc	f__Prevote	g__Prevote	s__pallens	ID1033790	1
k__Bacteri	p__Proteob	c__Gamma	o__Xantho	f__Xanthor	g__Pseudo	s__mexicar	ID104184	0
k__Bacteri	p__Firmicu	c__Bacilli	o__Lactoba	f__Strepto	g__Strepto	s__anginos	ID1047674	1
k__Bacteri	p__Firmicu	c__Bacilli	o__Lactoba	f__Strepto	g__Strepto	s__agalacti	ID1059977	u
k__Bacteri	p__Firmicu	c__Clostrid	o__Clostric	f__Peptost	g__Peptost	s__anaerob	ID105998	0
k__Bacteri	p__Proteob	c__Alphapr	o__Rhizobi	f__Methylc	g__Methyl	s__adhaesi	ID1074248	0
k__Bacteri	p__Proteob	c__Deltapr	o__Desulfo	f__Desulfol	g__Desulfo	s__rhabdof	ID10829	0
k__Bacteri	p__Firmicu	c__Clostrid	o__Clostric	f__Veillone	g__Selenor	s__noxia	ID1086250	6
k__Bacteri	p__Proteob	c__Betapro	o__Neisser	f__Neisseri	g__Neisser	s__bacillifo	ID1106060	0
k__Bacteri	p__Bacterc	c__Flavoba	o__Flavoba	f__Flavoba	g__Flavoba	s__gelidilac	ID1107152	0
k__Bacteri	p__Proteob	c__Gamma	o__Pseudo	f__Pseudor	g__Pseudo	s__alcalige	ID1107697	0
k__Bacteri	p__Synergi	c__Synergi	o__Synergi	f__Dethios	g__Pyramic	s__piscoler	ID1120396	0
k__Bacteri	p__Actinob	c__Actinob	o__Actinor	f__Dermab	g__Devries	s__agamar	ID1126230	0
k__Bacteri	p__Actinob	c__Actinob	o__Actinor	f__Micrococ	g__Rothia	s__terrae	ID1128355	2
k__Bacteri	p__Fusoba	c__Fusobac	o__Fusoba	f__Leptotri	g__Strepto	s__monilifo	ID11292	0
k__Bacteri	p__Firmicu	c__Bacilli	o__Lactoba	f__Enteroc	g__Enteroc	s__haemop	ID1129461	0
k__Bacteri	p__Firmicu	c__Clostrid	o__Clostric	f__Clostridi	g__Protein	s__ethanol	ID1133895	0
k__Bacteri	p__Proteob	c__Gamma	o__Xantho	f__Xanthor	g__Pseudo	s__kaohsiu	ID1135553	0
k__Bacteri	p__Bacterc	c__Flavoba	o__Flavoba	f__Flavoba	g__Zhouia	s__amyloly	ID1136009	3
k__Bacteri	p__Proteob	c__Betapro	o__Burkho	f__Comam	g__Aquinco	s__tertiaric	ID1137566	0
k__Bacteri	p__Actinob	c__Actinob	o__Actinor	f__Mycoba	g__Mycoba	s__vaccae	ID113826	0
k__Bacteri	p__Bacterc	c__Flavoba	o__Flavoba	f__Flavoba	g__Lutaone	s__thermo	ID1138276	0
k__Bacteri	p__Proteob	c__Gamma	o__Cardiob	f__Cardiob	g__Suttone	s__ornitho	ID1139148	0
k__Bacteri	p__Proteob	c__Alphapr	o__Sphingo	f__Sphingo	g__Sphingo	s__mali	ID1139751	1
k__Bacteri	p__Actinob	c__Corioba	o__Corioba	f__Corioba	g__Paraegg	s__hongko	ID1140324	0
k__Bacteri	p__Proteob	c__Gamma	o__Alteron	f__[Chroma	g__Rheinhe	s__perlucic	ID1141610	0
k__Bacteri	p__Actinob	c__Actinob	o__Actinor	f__Nocardi	g__Marmo	s__aequore	ID1142974	0
k__Bacteri	p__Firmicu	c__Clostrid	o__Clostric	f__Veillone	g__Selenor	s__bovis	ID1143338	13
k__Bacteri	p__Proteob	c__Gamma	o__Alteron	f__Shewan	g__Shewar	s__algae	ID115224	2
k__Bacteri	p__Actinob	c__Actinob	o__Actinor	f__Actinor	g__Actinon	s__hyovagi	ID12569	7
k__Bacteri	p__Proteob	c__Epsilon	o__Campyl	f__Helicob	g__Wolinel	s__succino	ID128801	0
k__Bacteri	p__Actinob	c__Actinob	o__Actinor	f__Coryneb	g__Corynel	s__pilosum	ID13467	0

k__Bacteria	p__Proteob	c__Alphapr	o__Rhizobi	f__Rhizobia	g__Shinella	s__granuli	ID136134	0
k__Bacteria	p__Proteob	c__Gamma	o__Xantho	f__Xanthor	g__Pseudo	s__indica	ID146193	0
k__Bacteria	p__Firmicu	c__Bacilli	o__Lactoba	f__Strepto	g__Strepto	s__minor	ID150451	0
k__Bacteria	p__Firmicu	c__Bacilli	o__Lactoba	f__Aerococ	g__Granuli	s__balaenc	ID15059	4
k__Bacteria	p__Proteob	c__Gamma	o__Oceanc	f__Halomo	g__Halomc	s__phocea	ID153241	1
k__Bacteria	p__Firmicu	c__Bacilli	o__Lactoba	f__Strepto	g__Strepto	s__equi	ID15355	0
k__Bacteria	p__Proteob	c__Betaprc	o__Trembl	f__Trembla	g__Carsone	s__ruddii	ID1543534	1
k__Bacteria	p__Bacterc	c__Bactero	o__Bacterc	f__Porphyr	g__Macelli	s__ferment	ID158646	1
k__Bacteria	p__Verrucc	c__Verrucc	o__Verrucc	f__Verruco	g__Verrucc	s__spinosu	ID1596	0
k__Bacteria	p__Proteob	c__Betaprc	o__Burkho	f__Comam	g__Comam	s__terriger	ID1639373	0
k__Bacteria	p__Firmicu	c__Bacilli	o__Lactoba	f__Strepto	g__Strepto	s__sobrinu	ID167118	0
k__Bacteria	p__Proteob	c__Gamma	o__Enterob	f__Enterob	g__Shigella	s__boydii	ID1780074	0
k__Bacteria	p__Proteob	c__Betaprc	o__Burkho	f__Comam	g__[Polyan	s__brachys	ID181669	0
k__Bacteria	p__Proteob	c__Gamma	o__Pseudo	f__Moraxe	g__Acineto	s__venetia	ID181761	0
k__Bacteria	p__Firmicu	c__Bacilli	o__Lactoba	f__Lactoba	g__Lactoba	s__mucosa	ID1911770	0
k__Bacteria	p__Actinob	c__Actinob	o__Actinor	f__Micrococ	g__Rothia	s__amarae	ID1925093	0
k__Bacteria	p__Bacterc	c__Bactero	o__Bacterc	f__Porphyr	g__Porphyı	s__endodo	ID1938	1
k__Bacteria	p__Proteob	c__Gamma	o__Alteron	f__Shewan	g__Shewar	s__amazon	ID197265	0
k__Bacteria	p__Bacterc	c__Bactero	o__Bacterc	f__[Parapre	g__[Prevot	s__tannera	ID2047	5
k__Bacteria	p__Bacterc	c__Bactero	o__Bacterc	f__Prevote	g__Prevote	s__interme	ID2218	25
k__Bacteria	p__Proteob	c__Gamma	o__Pseudo	f__Moraxe	g__Moraxe	s__ovis	ID223485	0
k__Bacteria	p__Spiroch	c__Spiroch	o__Spiroch	f__Spiroch	g__Trepon	s__socrans	ID2310825	2
k__Bacteria	p__Actinob	c__Actinob	o__Actinor	f__Tsukam	g__Gordon	s__kropper	ID236778	0
k__Bacteria	p__Proteob	c__Alphapr	o__Rhizobi	f__Methylc	g__Magnet	s__magnet	ID237209	0
k__Bacteria	p__Actinob	c__Actinob	o__Actinor	f__Beutenb	g__Beutenl	s__caverna	ID2500552	0
k__Bacteria	p__Actinob	c__Corioba	o__Corioba	f__Corioba	g__Atopob	s__rimae	ID251702	0
k__Bacteria	p__Proteob	c__Betaprc	o__Burkho	f__Comam	g__Brachyr	s__chirono	ID265983	0
k__Bacteria	p__Elusimi	c__Elusimic	o__Elusimi	f__Elusimic	g__Elusimi	s__minutur	ID278595	0
k__Bacteria	p__Plancto	c__Plancto	o__Pirellula	f__Pirellula	g__plancto	s__MS1399	ID2905450	0
k__Bacteria	p__Spiroch	c__Spiroch	o__Spiroch	f__Spiroch	g__Trepon	s__amylov	ID29328	0
k__Bacteria	p__Firmicu	c__Clostrid	o__Clostric	f__Veillone	g__Aceton	s__longum	ID3107842	0
k__Bacteria	p__Bacterc	c__Flavoba	o__Flavoba	f__Flavoba	g__Capnoc	s__canimoı	ID314128	0
k__Bacteria	p__Proteob	c__Gamma	o__Alteron	f__[Chroma	g__Rheinhe	s__nanhaie	ID325282	0
k__Bacteria	p__Teneric	c__Mollicu	o__Mycopl	f__Mycopla	g__Mycopl	s__zalophi	ID336960	0
k__Bacteria	p__Bacterc	c__Bactero	o__Bacterc	f__Bactero	g__Bacterc	s__nordii	ID344827	0
k__Bacteria	p__Firmicu	c__Bacilli	o__Lactoba	f__Leucon	g__Weissel	s__parame	ID352468	0
k__Bacteria	p__Firmicu	c__Bacilli	o__Bacillal	f__Planoco	g__Staphyl	s__saproph	ID354839	0
k__Bacteria	p__Proteob	c__Gamma	o__Pasteur	f__Pasteur	g__Gallibac	s__genomc	ID359719	0
k__Bacteria	p__Actinob	c__Actinob	o__Bifidob	f__Bifidoba	g__Bifidob	s__bifidum	ID365385	0
k__Bacteria	p__Spiroch	c__Spiroch	o__Spiroch	f__Spiroch	g__Trepon	s__phaged	ID3928	0
k__Bacteria	p__Proteob	c__Gamma	o__Aeromc	f__Aeromo	g__Aeromc	s__sanarell	ID4006670	0
k__Bacteria	p__Firmicu	c__Erysipel	o__Erysipe	f__Erysipel	g__Bulleidi	s__moorei	ID4030370	0
k__Bacteria	p__Proteob	c__Gamma	o__Pseudo	f__Moraxe	g__Enhydr	s__aerosac	ID407441	0
k__Bacteria	p__Proteob	c__Epsilon	o__Campyl	f__Campyl	g__Campyl	s__rectus	ID416537	15
k__Bacteria	p__Proteob	c__Betaprc	o__Neisser	f__Neisseri	g__Neisser	s__shayega	ID4233669	0
k__Bacteria	p__Proteob	c__Gamma	o__Vibrion	f__Pseudoa	g__Vibrio	s__mimicus	ID4270835	0
k__Bacteria	p__Bacterc	c__Flavoba	o__Flavoba	f__[Weeks	g__Bergeye	s__zoohelc	ID4297519	1
k__Bacteria	p__Proteob	c__Gamma	o__Pseudo	f__Pseudor	g__Pseudo	s__cuatroc	ID4301996	0
k__Bacteria	p__Proteob	c__Gamma	o__Xantho	f__Xanthor	g__Stenotr	s__rhizoph	ID4331923	0
k__Bacteria	p__Firmicu	c__Clostrid	o__Clostric	f__Lachnos	g__Johnsor	s__ignava	ID4332505	0

k__Bacteria	p__Proteob	c__Betapro	o__Neisser	f__Neisseri	g__Neisser	s__wadswo	ID4335663	0
k__Bacteria	p__Proteob	c__Betapro	o__Neisser	f__Neisseri	g__Neisser	s__weaveri	ID4337487	1
k__Bacteria	p__Fusoba	c__Fusobac	o__Fusoba	f__Leptotri	g__Sebalde	s__termitic	ID4358870	15
k__Bacteria	p__Proteob	c__Alphapr	o__Rhizobi	f__Bradyrh	g__Afipia	s__clevelar	ID4367627	0
k__Bacteria	p__Firmicu	c__Clostrid	o__Clostrid	f__Clostridi	g__Clostrid	s__caminitl	ID4368040	0
k__Bacteria	p__Proteob	c__Betapro	o__Neisser	f__Neisseri	g__Neisser	s__oralis	ID4373910	368
k__Bacteria	p__Proteob	c__Betapro	o__Burkho	f__Comam	g__Leptoth	s__ginseng	ID4395318	0
k__Bacteria	p__Firmicu	c__Clostrid	o__Clostrid	f__Clostridi	g__Clostrid	s__aciduric	ID4462654	0
k__Bacteria	p__Proteob	c__Betapro	o__Neisser	f__Neisseri	g__Kingella	s__potus	ID4476050	0
k__Bacteria	p__Synergi	c__Synergi	o__Synergi	f__Dethios	g__Jonque	s__anthrop	ID484439	0
k__Bacteria	p__Firmicu	c__Clostrid	o__Clostrid	f__Lachnos	g__Moryell	s__indolige	ID514200	1
k__Bacteria	p__Plancto	c__Plancto	o__Gemma	f__Isospha	g__Nostoc	s__limicola	ID51778	0
k__Bacteria	p__Proteob	c__Gamma	o__Cardiob	f__Cardiob	g__Cardiob	s__valvarui	ID544152	8
k__Bacteria	p__Proteob	c__Alphapr	o__Rhizobi	f__Beijerin	g__Chelato	s__asaccha	ID548469	0
k__Bacteria	p__Proteob	c__Gamma	o__Pseudo	f__Moraxe	g__Alkanin	s__illinoise	ID552246	0
k__Bacteria	p__Proteob	c__Gamma	o__Enterob	f__Enterob	g__Serratia	s__symbiot	ID576678	0
k__Bacteria	p__Proteob	c__Betapro	o__Neisser	f__Neisseri	g__Neisser	s__elongat	ID6402	3
k__Bacteria	p__Proteob	c__Gamma	o__Pseudo	f__Pseudor	g__Pseudo	s__compos	ID673640	0
k__Bacteria	p__Firmicu	c__Bacilli	o__Lactoba	f__Aerococ	g__Abiotro	s__defectiv	ID678690	0
k__Bacteria	p__Firmicu	c__Clostrid	o__Clostrid	f__Veillone	g__Anaero	s__glycerin	ID731367	6
k__Bacteria	p__Firmicu	c__Erysipel	o__Erysipe	f__Erysipel	g__Anaero	s__furcosa	ID761476	0
k__Bacteria	p__Proteob	c__Alphapr	o__Rhodos	f__Rhodos	g__Reyran	s__massilie	ID801271	0
k__Bacteria	p__Proteob	c__Alphapr	o__Rhodos	f__Acetoba	g__Acidispl	s__rubrifac	ID806916	0
k__Bacteria	p__Proteob	c__Gamma	o__Pseudo	f__Pseudor	g__Pseudo	s__alcaliph	ID83866	0
k__Bacteria	p__Firmicu	c__Clostrid	o__Clostrid	f__Lachnos	g__Shuttle	s__satelles	ID851782	0
k__Bacteria	p__Proteob	c__Betapro	o__Neisser	f__Neisseri	g__Kingella	s__denitrifi	ID851932	29
k__Bacteria	p__Proteob	c__Gamma	o__Pasteur	f__Pasteur	g__Pasteur	s__aeroger	ID9498	0
k__Bacteria	p__Proteob	c__Gamma	o__Pasteur	f__Pasteur	g__Actinob	s__delphin	ID9510	0

tes. The high quality seuences were processed through Mothur package against gg_13_8_99.gg.tax

CP10	CP11	CP12	CP13	CP14	CP15	CP16	CP17	CP18
0	0	0	0	0	0	0	1	0
0	0	0	0	0	0	0	0	0
14	6	15	1	8	22	25	1	3
103	8	11	109	5	258	225	23	194
0	0	0	0	0	0	0	0	0
0	0	0	0	0	0	0	0	0
0	0	0	0	0	0	0	0	0
0	0	0	0	0	0	0	0	0
0	0	0	0	0	0	0	0	0
0	0	0	1	0	0	0	0	2
0	0	0	0	0	0	0	0	0
65	60	150	3	64	29	12	0	11
23	10	23	78	42	194	69	30	46
0	0	0	0	0	0	0	0	0
1	0	0	0	0	0	0	0	0
0	5	14	0	0	0	1	1	2
0	0	0	0	0	0	0	0	0
62	0	0	0	0	0	2	0	0
7	12	15	166	25	25	91	38	38
13	19	42	0	0	0	1	5	1
0	0	0	0	0	0	0	0	0
17	7	6	0	0	0	6	4	1
2	7	10	19	89	307	4	1	0
0	0	0	0	0	0	0	0	13
0	0	0	0	0	0	0	0	0
0	0	0	0	0	0	0	0	0
18	3	13	0	0	0	2	0	0
0	0	0	0	0	0	0	0	0
2	0	1	3	2	5	0	1	15
0	0	0	0	0	0	0	0	0
0	0	0	0	0	0	0	0	0
0	0	0	0	0	0	0	0	0
0	0	0	0	0	0	0	0	0
1	0	0	4	2	0	1	0	2
0	0	0	0	0	0	0	0	0
0	0	0	0	0	0	0	0	0
0	1	0	0	0	0	0	0	0
0	0	0	0	0	0	0	0	0
0	2	2	0	0	0	3	1	2
0	0	0	0	0	0	0	0	0
0	0	0	0	0	0	0	0	0
0	0	0	0	0	0	0	0	0
235	95	148	21	83	132	8	22	6
1	2	1	0	0	0	0	1	2
0	1	2	9	5	16	3	0	14
0	0	0	0	0	0	0	0	0
0	1	0	0	0	3	0	0	0

0	0	0	0	0	0	0	0	0
0	0	0	0	0	0	0	0	0
0	0	0	0	0	0	0	0	0
0	1	6	23	1	9	18	5	5
3	8	7	1	1	5	3	6	0
0	0	0	0	0	0	0	0	0
2	5	5	63	1	5	5	1	7
84	275	669	3	28	10	80	95	45
0	0	0	0	0	0	0	0	0
0	0	0	0	0	0	0	0	0
0	0	0	0	0	0	0	0	13
0	0	0	0	0	0	0	0	0
0	0	0	0	0	0	0	0	0
0	0	0	0	0	0	0	0	0
0	0	0	0	1	0	0	0	0
0	0	0	0	0	0	0	0	0
46	12	25	0	1	1	18	59	13
0	0	0	0	0	0	1	0	0
48	57	105	0	15	5	13	15	3
82	1	4	0	8	1	5	384	89
0	0	0	0	0	0	0	0	0
28	28	58	0	11	3	1	18	1
0	0	0	0	0	0	0	0	0
0	0	0	0	0	0	0	0	0
0	0	0	0	0	0	0	0	0
0	0	3	0	5	1	2	0	0
4	2	4	0	0	0	3	0	0
0	0	0	0	0	0	0	1	0
0	0	0	0	0	0	0	0	0
11	0	0	0	0	0	1	36	2
0	0	0	0	0	0	0	0	0
0	0	0	0	0	1	0	0	0
0	0	0	0	0	0	0	0	0
0	1	0	0	0	0	0	4	0
1	0	7	0	0	0	0	3	0
0	0	0	0	0	0	0	0	0
0	0	0	0	0	0	0	0	0
0	0	0	0	0	0	0	0	0
0	0	0	0	0	0	0	0	0
10	22	37	1	11	1	5	22	6
0	0	0	0	0	0	0	0	0
1	4	4	0	3	0	1	0	0
0	0	0	0	0	0	0	0	0
144	34	48	1	16	56	41	15	4
0	0	0	0	0	0	0	0	0
0	0	0	0	0	0	0	0	0
0	1	1	9	0	0	5	2	0
0	0	0	0	0	0	0	0	0
0	0	0	0	0	0	0	0	0
10	3	4	1	0	2	1	13	0

0	0	0	0	0	0	0	0	0
0	0	0	0	1	4	0	0	2
10	15	22	6	64	92	53	5	16
0	0	0	0	0	0	0	0	0
4	0	1	0	0	0	1	4	0
80	7	8	812	20	95	716	378	67
0	0	0	0	0	0	0	0	0
1	0	0	0	0	0	0	1	0
0	0	0	0	0	0	0	0	0
0	0	0	0	0	0	0	0	0
1	0	0	0	1	0	0	0	0
0	0	0	0	0	0	0	0	0
0	0	3	0	2	1	3	2	14
0	0	0	0	0	0	0	0	0
0	1	0	0	0	0	1	0	0
0	0	0	0	0	0	0	0	0
0	2	3	0	1	7	1	0	2
0	0	0	0	0	0	0	0	0
0	0	2	67	1	2	18	34	7
11	2	3	17	1	21	40	2	29
0	0	1	0	0	0	0	0	0
4	4	0	1	4	0	2	2	5
0	0	0	0	0	0	0	0	0
0	0	0	0	0	0	0	0	0
0	1	0	0	3	0	0	0	0
0	0	0	21	1	0	2	48	14
0	0	1	0	0	2	0	0	0
0	0	0	0	0	0	0	0	0

CP19	CP2	CP20	CP21	CP22	CP23	CP24	CP26	CP27
0	0	0	0	0	0	2	0	0
0	6	0	1	0	0	0	0	5
4	2	14	1	6	7	4	11	2
20	0	15	7	158	13	188	50	29
0	0	0	0	0	0	0	0	0
0	0	0	0	0	0	0	0	0
0	0	0	0	0	0	3	0	0
0	0	0	0	0	0	2	0	0
0	0	0	0	0	0	0	0	0
0	0	0	0	0	0	0	0	0
0	0	0	0	0	0	0	0	0
27	66	15	1	17	77	45	12	18
108	2	44	623	332	47	137	192	44
0	0	0	0	0	0	0	0	0
0	0	0	0	0	0	0	0	0
0	0	0	0	0	0	3	0	10
0	0	0	0	0	0	0	0	0
0	0	0	0	0	0	0	1	0
75	3	28	91	25	18	65	39	30
1	0	0	0	1	9	4	2	0
0	0	0	0	0	0	0	0	0
5	6	1	0	0	0	0	0	1
0	5	0	0	25	68	5	5	7
2	5	0	40	0	1	1	0	1
0	0	0	0	0	0	0	0	0
0	0	0	0	0	0	0	0	0
0	0	0	0	0	0	0	27	0
0	0	0	0	0	0	0	0	0
9	0	0	4	6	1	10	0	2
0	0	0	0	0	0	0	0	0
0	0	0	0	0	0	0	0	0
0	0	0	0	0	0	0	0	0
0	0	0	0	0	0	0	0	0
6	0	5	1	1	0	5	6	0
0	0	0	0	0	0	0	0	0
0	0	0	0	0	0	0	0	0
0	0	0	0	0	0	0	0	0
0	0	0	0	1	0	0	0	0
1	0	0	0	1	0	7	0	1
0	0	1	0	0	0	0	0	0
0	0	0	0	0	0	0	0	0
0	0	0	0	0	0	0	0	0
33	9	94	0	15	66	3	24	44
1	0	0	0	0	1	3	1	0
15	0	12	0	12	3	11	4	0
0	1	0	0	0	0	0	0	0
0	0	2	0	0	1	2	0	0

0	0	0	0	0	0	0	0	0
0	0	0	0	0	0	0	0	0
0	0	0	0	0	0	0	0	0
10	2	3	41	6	4	53	3	10
3	1	0	0	0	4	11	0	0
1	0	0	0	1	0	0	0	0
2	3	4	28	10	4	10	3	11
32	22	33	1	0	121	5	29	58
0	0	0	0	0	0	0	0	0
0	0	0	0	0	0	0	0	0
0	0	0	0	0	0	7	0	0
0	0	0	0	0	0	0	0	0
0	0	0	0	0	0	0	0	0
0	0	0	0	0	0	0	0	0
0	0	0	0	0	0	0	0	0
0	0	0	0	0	1	1	0	0
15	8	40	2	0	46	1	4	1
0	0	0	0	0	1	1	0	0
0	24	10	0	0	6	1	60	0
77	35	61	1	3	1	4	2	42
0	0	0	0	0	0	0	0	0
5	12	13	0	0	6	1	12	1
0	0	0	0	0	0	0	0	0
0	0	0	0	0	0	0	0	0
0	0	0	0	0	0	0	0	0
3	0	1	0	2	5	2	7	1
0	0	1	0	0	1	0	0	0
0	0	0	0	0	0	0	0	0
0	0	0	0	0	0	0	0	0
10	5	9	0	0	0	1	2	1
0	0	0	0	0	0	0	0	0
1	0	1	3	0	0	0	0	0
0	0	0	0	0	0	0	0	0
1	1	0	0	0	0	0	0	0
1	5	1	0	0	3	0	0	2
0	0	0	0	0	0	0	0	0
0	0	0	0	0	0	0	0	0
0	0	0	0	0	0	0	0	0
0	0	0	0	0	2	0	0	0
7	5	9	0	0	16	1	2	2
0	0	0	0	0	0	0	0	0
0	0	0	0	3	9	6	0	0
0	0	0	0	0	0	0	0	0
24	50	24	14	43	47	6	12	36
0	1	0	1	0	0	1	0	1
0	0	0	0	0	0	0	0	0
8	0	1	1	1	1	9	8	13
0	0	0	0	0	0	0	0	0
0	0	0	0	0	0	0	0	0
4	0	6	0	1	11	0	0	3

0	0	0	0	0	0	0	0	0
1	10	0	0	0	1	6	0	0
5	26	18	21	3	12	19	13	25
0	0	0	0	0	0	0	0	0
0	0	1	0	2	1	0	0	2
238	12	28	1800	66	12	138	163	735
0	0	0	0	0	0	0	0	0
0	0	0	0	0	1	0	0	0
0	0	0	0	0	0	0	0	1
0	0	0	0	0	0	0	0	0
0	0	0	0	1	2	1	0	0
0	0	0	0	0	0	0	0	0
2	11	12	0	2	2	21	0	2
0	0	0	0	0	0	0	0	0
0	0	0	0	0	0	2	0	0
0	0	0	0	0	0	0	3	0
14	7	10	0	1	1	0	1	0
0	0	0	0	0	0	0	0	0
5	0	3	101	0	0	3	2	11
13	0	8	1	19	12	17	19	14
0	0	0	0	0	0	0	0	0
2	0	0	2	2	1	16	0	1
0	0	0	0	0	0	0	0	0
0	0	0	0	0	0	0	0	0
0	0	0	0	1	12	0	0	0
10	6	10	3	0	1	61	3	13
0	0	0	0	0	0	0	1	0
0	0	0	0	0	0	0	0	0

CP3	CP30	CP31	CP32	CP33	CP34	CP35	CP36	CP37
0	0	0	0	0	0	0	0	0
0	0	0	12	0	0	0	0	0
12	16	1	16	0	4	0	17	5
0	23	1	1	0	1	18	28	4
0	0	0	0	0	0	0	0	0
0	0	0	0	0	0	1	0	0
0	0	0	0	0	0	0	0	0
0	0	0	0	0	0	0	0	0
0	0	0	0	0	0	0	0	0
0	0	0	0	0	0	0	0	0
0	0	0	0	0	0	0	0	0
2	45	3	0	8	1	0	0	9
71	4	3	4	20	39	51	54	3
0	0	0	0	0	0	0	0	0
0	0	0	0	0	0	0	0	0
0	8	0	0	0	0	0	0	0
0	0	0	0	0	0	0	0	0
0	0	0	0	0	0	0	0	0
17	64	5	28	3	32	5	31	12
1	2	0	0	3	0	0	0	8
0	0	0	0	0	0	0	0	0
4	0	0	0	1	0	0	9	2
0	6	74	0	2	0	0	0	1
0	0	0	0	2	0	0	0	12
0	0	0	0	0	0	0	0	0
0	0	0	0	0	0	0	0	0
0	0	0	0	0	0	0	0	0
0	0	0	0	0	0	0	0	0
2	1	0	0	0	0	0	3	2
0	0	0	0	0	0	0	0	0
0	0	0	0	0	0	0	0	0
0	0	0	0	0	0	0	0	0
0	0	0	0	0	0	0	0	0
6	0	1	11	1	2	0	3	1
0	0	0	0	0	0	0	0	0
0	0	0	0	0	0	0	0	0
0	0	0	0	0	0	0	0	0
0	0	0	0	0	0	0	0	0
2	0	1	0	0	0	0	1	0
0	0	0	0	0	0	0	0	0
0	0	0	0	0	0	0	0	0
0	0	0	0	0	0	0	0	0
14	45	24	0	13	1	2	40	117
1	0	0	0	0	0	0	0	0
0	0	0	0	0	0	0	9	0
0	0	0	0	0	0	1	0	0
0	0	0	0	0	0	0	0	0

0	0	0	0	0	0	0	0	0
0	0	0	0	0	0	0	0	0
0	0	0	0	0	0	0	0	0
4	2	0	1	1	14	12	1	1
1	0	0	0	0	0	0	0	0
1	0	0	0	0	0	0	0	0
47	1	0	31	5	0	0	1	8
104	3	1	0	3	1	0	9	22
0	0	0	0	0	0	0	0	0
0	0	0	0	0	0	0	0	0
0	0	0	0	0	0	0	0	0
0	0	0	0	0	0	0	0	0
0	0	0	0	0	0	0	0	0
0	0	0	0	0	0	0	0	0
0	0	0	0	0	0	0	0	0
0	0	0	0	0	0	0	0	0
54	7	2	0	3	0	1	1	28
0	0	0	0	0	0	0	0	0
67	0	0	0	2	0	0	3	4
43	23	8	0	16	1	1	6	137
0	2	0	0	0	0	0	0	0
12	0	0	0	7	0	0	0	4
0	0	0	0	0	0	0	0	0
0	0	0	0	0	0	0	0	0
0	0	0	0	0	0	0	0	0
0	1	0	0	1	0	0	0	9
0	0	0	0	0	0	0	1	0
0	0	0	0	0	0	0	0	0
0	0	0	0	0	0	0	0	0
0	0	0	0	0	0	0	0	1
0	0	0	0	0	0	0	0	0
0	0	0	0	0	0	0	0	0
0	0	0	0	0	0	0	0	0
2	0	0	0	0	0	0	0	0
0	0	0	0	0	0	0	0	1
0	0	0	0	0	0	0	0	0
0	0	0	0	0	0	0	0	0
0	0	0	0	0	0	1	0	0
0	0	0	0	0	0	0	0	1
53	0	0	0	0	0	0	1	0
0	0	0	0	0	0	0	0	0
0	0	0	0	0	0	0	0	1
0	0	0	0	0	0	0	0	0
29	12	2	11	20	3	0	22	25
0	0	0	0	0	0	0	0	0
0	0	0	0	0	0	0	0	0
7	1	0	3	1	9	1	0	2
0	0	0	0	0	0	0	0	0
0	0	0	0	0	0	0	0	0
0	0	0	0	2	0	0	0	1

0	0	0	0	0	0	0	0	0
3	0	0	0	3	2	1	1	3
29	7	0	2	4	2	0	3	3
0	0	0	0	0	0	0	0	0
0	0	0	0	1	0	0	0	1
1251	122	1	1260	72	233	63	110	6
0	0	0	0	0	0	0	0	0
0	0	0	0	0	0	0	0	0
0	0	0	0	0	0	0	0	0
0	0	0	0	0	0	0	0	0
0	0	0	0	0	0	0	0	0
0	0	0	0	0	0	0	0	0
0	0	1	0	0	0	0	0	0
0	0	0	0	0	0	0	0	0
0	0	0	0	0	0	0	0	0
0	0	0	0	0	0	0	0	0
34	1	1	1	8	10	0	58	13
0	0	0	0	0	0	0	0	0
1	0	0	6	0	0	11	5	0
2	24	1	1	0	2	5	12	3
0	0	0	0	0	0	0	0	2
4	0	0	0	0	0	0	1	0
0	0	0	0	0	0	0	0	0
0	0	0	0	0	0	0	0	0
0	1	0	0	0	0	0	1	8
19	2	1	6	8	3	0	19	2
0	0	0	1	0	0	0	0	2
1	0	0	0	0	0	0	0	0

CP38	CP39	CP4	CP40	CP5	CP6	CP7	CP8	CP9
0	0	0	0	8	0	0	0	0
0	1	0	0	15	0	0	0	0
18	2	1	4	64	2	12	4	2
42	0	1	34	29	363	17	52	3
0	0	0	0	0	0	0	0	0
0	0	0	0	0	0	0	0	0
0	0	0	0	0	0	0	0	0
0	0	0	0	5	0	0	0	0
2	0	0	0	0	0	0	0	0
0	0	0	0	0	0	0	0	0
0	0	0	0	2	0	0	0	0
51	7	0	0	0	24	64	32	0
35	21	93	4	219	12	49	98	244
0	0	0	0	2	0	0	0	0
0	0	0	0	0	0	0	0	0
0	7	0	0	258	0	0	0	0
0	0	0	0	153	0	0	0	0
0	0	0	0	0	0	1	1	2
25	14	19	35	37	80	6	11	9
0	6	0	1	0	1	0	0	1
0	0	0	0	0	0	0	0	0
1	0	0	5	0	0	10	0	0
24	3	0	0	0	0	4	282	8
2	4	1	0	0	0	0	0	0
0	0	0	0	1	0	0	0	0
0	0	0	0	8	0	0	0	0
0	0	0	0	0	0	0	0	0
0	0	0	0	0	0	0	0	0
1	0	1	12	0	7	5	10	25
0	0	0	0	1	0	0	0	0
0	0	0	2	1	0	0	0	0
0	0	0	0	0	0	1	0	0
0	0	0	0	72	0	0	0	0
5	1	15	0	0	0	4	1	6
0	0	0	0	3	0	0	0	0
0	0	0	0	1	0	0	0	0
0	0	0	0	0	0	0	0	0
0	0	0	0	0	0	0	0	0
1	0	0	1	16	0	2	0	1
0	0	0	0	0	0	0	0	0
0	0	0	0	9	0	0	0	0
0	0	0	0	1	0	0	0	0
39	82	1	3	129	1	34	103	14
0	0	0	0	97	0	2	0	0
0	0	0	0	9	5	0	2	2
0	0	0	0	0	0	0	0	0
3	0	0	0	0	0	0	8	0

0	0	0	0	1	0	0	0	0
0	0	0	0	11	0	0	0	0
0	0	0	0	0	0	0	0	0
8	2	2	2	74	8	2	0	0
0	0	0	0	235	3	1	2	2
0	0	1	2	0	3	0	0	1
0	5	11	5	12	6	8	21	3
13	4	18	135	2	3	451	2	49
0	0	0	0	3	0	0	0	0
0	0	0	0	12	0	0	0	0
0	0	0	0	0	0	0	0	0
0	0	0	0	1	0	0	0	0
0	0	0	0	19	0	0	0	0
0	0	0	0	4	0	0	0	0
0	0	0	0	0	0	0	1	0
0	0	0	0	0	0	0	0	0
10	45	10	4	0	4	0	0	27
0	0	0	0	3	0	0	0	0
14	39	0	0	0	1	0	36	6
22	346	6	7	28	6	1	8	4
0	0	0	0	0	0	0	0	0
3	8	5	4	0	0	108	3	3
0	0	0	0	2	0	0	0	0
0	0	0	0	5	0	0	0	1
0	0	0	0	0	0	0	1	0
3	4	0	0	1	0	2	2	0
0	0	0	0	0	0	0	0	0
0	0	0	0	0	0	0	0	0
0	0	0	0	3	0	0	0	0
0	3	0	3	0	0	0	0	1
0	0	0	0	0	0	0	0	0
1	0	2	0	1	0	1	0	0
0	0	0	0	0	0	0	0	0
0	0	0	1	0	0	0	0	0
2	2	2	0	5	1	0	0	0
0	0	0	0	1	0	0	0	0
0	0	0	0	1	2	0	0	0
0	0	0	0	0	0	0	0	0
0	0	0	0	0	0	0	1	0
5	1	10	13	0	1	0	0	23
0	0	0	0	1	0	0	0	0
1	0	0	0	1	1	0	0	0
0	0	0	0	0	0	0	0	0
19	55	10	51	10	5	83	34	32
0	0	0	0	0	0	0	0	0
0	0	0	0	2	0	0	0	0
6	53	1	0	3	0	1	0	1
0	0	0	0	1	0	0	0	0
0	0	0	0	1	0	0	0	0
0	3	0	1	0	0	0	0	3

0	0	0	0	0	0	1	0	0
19	0	0	0	2	0	12	2	0
17	11	22	1	131	4	54	10	19
0	0	0	0	9	0	0	0	0
0	1	0	1	3	0	0	0	1
27	21	782	0	322	447	56	27	1
0	0	0	0	8	0	0	0	0
0	0	0	0	0	0	0	0	0
0	0	0	0	0	0	0	0	0
0	0	0	4	0	0	0	0	0
0	0	0	0	14	0	0	0	1
0	0	0	0	2	0	0	0	0
3	2	0	0	0	0	0	0	0
0	0	0	0	2	0	0	0	0
0	0	0	0	4	0	0	0	0
0	0	0	0	0	0	0	0	0
6	5	0	0	0	0	7	0	0
0	0	0	0	8	0	0	0	0
1	3	0	0	2	0	0	0	0
12	0	1	23	22	88	3	7	6
0	0	0	0	0	0	0	0	0
0	0	0	0	0	0	1	0	0
0	0	0	0	5	0	0	0	0
0	0	0	0	4	0	0	0	0
0	0	0	0	0	0	0	0	0
3	4	27	2	1	0	4	0	0
0	1	0	0	0	0	0	0	0
0	0	0	0	0	0	0	1	0

Total OTUs
11
41
329
2072
0
2
3
7
2
3
2
926
3177
2
1
310
153
70
1224
121
0
86
967
87
1
8
63
0
132
1
3
1
72
94
3
1
1
1
46
1
9
1
1717
115
141
2
20

1
11
0
338
298
10
334
2420
3
12
20
1
19
4
2
2
491
6
539
1491
3
357
2
6
1
56
16
1
3
86
0
11
0
10
36
1
3
1
4
264
1
35
0
1053
4
2
150
1
1
69

1
74
760
9
24
10557
8
3
1
4
22
2
91
2
8
3
197
8
286
447
3
53
5
4
27
324
0
0
